# Transcriptome analysis reveals autophagy as regulator of TGFβ/Smad-induced fibrogenesis in trabecular meshwork cells

**DOI:** 10.1038/s41598-019-52627-2

**Published:** 2019-11-06

**Authors:** April Nettesheim, Myoung Sup Shim, Josh Hirt, Paloma B. Liton

**Affiliations:** Duke University, Department of Ophthalmology, Durham, NC USA

**Keywords:** Macroautophagy, Extracellular signalling molecules

## Abstract

The trabecular meshwork (TM) is a specialized ocular tissue, which is responsible, together with the Schlemm’s canal (SC), for maintaining appropriate levels of intraocular pressure. Dysfunction of these tissues leads to ocular hypertension and increases the risk for developing glaucoma. Previous work by our laboratory revealed dysregulated autophagy in aging and in glaucomatous TM cells. In order to gain more insight in the role of autophagy in the TM pathophysiology, we have conducted transcriptome and functional network analyses of TM primary cells with silenced expression of the autophagy genes Atg5 and Atg7. Atg5/7-deficient TM cells showed changes in transcript levels of several fibrotic genes, including TGFβ2, BAMBI, and SMA. Furthermore, genetic and pharmacological inhibition of autophagy was associated with a parallel reduction in TGFβ-induced fibrosis, caused by a BAMBI-mediated reduced activation of Smad2/3 signaling in autophagy-deficient cells. At the same time, TGFβ treatment led to Smad2/3-dependent dysregulation of autophagy in TM cells, characterized by increased LC3-II levels and autophagic vacuoles content. Together, our results indicate a cross-talk between autophagy and TGFβ signaling in TM cells.

## Introduction

The trabecular meshwork (TM) is a specialized tissue in the anterior segment of the eye which, together with the Schlemm’s canal (SC), functions primarily to maintain proper intraocular pressure (IOP) levels. The functional decline of this tissue, in particular with aging, causes elevation in IOP. This increases the risk of developing glaucoma, the second leading cause of blindness worldwide^1^. The specific causes leading to dysfunction of the TM tissue are still not fully characterized and are believed to multifactorial, similar to other complex age-related diseases. Several morphological changes have been described in the TM from glaucoma patients in comparison to age-matched controls. These include decrease in cellularity, abnormal accumulation of extracellular matrix (ECM) and thickening of the trabecular beams^2,3^. In patients with glaucoma, increased levels of transforming growth factor-β2 (TGFβ2) in the aqueous humor (AH) have been also observed in numerous studies by different laboratories, which are believed to contribute to the increased ECM deposition and stiffness^4–6^. In addition to these, our group has reported increased activity of senescence-associated-β-galactosidase (SA-β-Gal) in glaucomatous TM cells both *in situ*^3^ and *in vitro*^7,8^, which was associated with dysregulated autophagy and diminished lysosomal proteolysis^7,8^. Decreased autophagic flux has been also more recently shown by our lab in the TM of DBA/2J mice, an ocular hypertensive mouse model closely resembling human pigmentary glaucoma^9^.

Autophagy is a process by which cellular components, including macromolecules and organelles, are degraded into their smallest constituents within lysosomes by the action of acidic hydrolases. In the best-studied form of autophagy, macroautophagy - therein after autophagy - cytoplasmic cargo is sequestered by the autophagosome, a newly-formed double-membraned organelle, which then can fuse with the lysosomes, forming the autolysosome, where content is degraded. The molecular mechanisms of autophagy in mammals have just lately been characterized. It involves several conserved autophagy-related (Atg) proteins and two ubiquitin-like conjugation pathways, catalyzed by Atg7, that result in lipidation of the autophagosome marker LC3-I into LC3-II, which is subsequently attached to both the inner and outer membrane of the expanding phagophore. In the autolysosome, LC3-II can be degraded or recycled into LC3-I. Autophagy occurs in all cell types at basal levels, as it is essential for the maintenance of cellular homeostasis, but it is quickly upregulated in response to several types of stress^10–12^. Dysregulated autophagy has been connected to an increasing number of human diseases, particularly in age- related diseases and neurodegenerative disorders^13–15^. However, the function of autophagy in disease seems to be highly pleiotropic. Autophagy has been revealed to be an adaptive pro-survival response in some instances, but in other conditions autophagy seems to stimulate cell death^16^.

In order to gain more insight in the role of dysregulated autophagy in the TM pathophysiology and glaucoma, we have explored the effects of downregulating autophagy on TM cell function. Our results indicate a cross-talk between autophagy and TGFβ signaling pathway, and a role of autophagy in regulating fibrosis in TM cells.

## Material and Methods

### Reagents

TGFβ1 (T7039), TGFβ2 (T2815), rapamycin (R8781), bafilomycin A1 (BafA1, B1793), and 3-methyladenine (3-MA, M9281) were all obtained from Sigma-Aldrich.

### Isolation and characterization of human TM cells

Human TM cells were isolated from dissected TM tissue from discarded corneal rims after surgical corneal transplantation at Duke University Eye Center, and distributed upon informed consent by BioSight (Duke Core Service), and cultured as described earlier^17^. Cells were passaged 1:2 when confluent and were used at passages 4–6 in this study. Cells were characterized as TM cells by observing morphology and by the upregulation of myocilin in response to dexamethasone treatment, in accordance to the consensus recommendations for TM cell isolation, characterization and culture^18^. The protocols involving the use of human tissue were approved under the Duke University Institutional Review Board (protocol #00050810) and were consistent with the tenets of the Declaration of Helsinki.

### siRNA transfection

siRNA transfection was performed as described previously with minor modifications^19^. In brief, primary human TM cells were plated on 24-well plate in 0.5 mL of the growth media, with 60–80% confluence. After 24 h, the cells were transfected with either 5 pmol of siRNA against ATG5 (siAtg5, sc-41445), ATG7 (siAtg7, sc-41447), LC3 (siLC3, sc-43391), Smad2/3 (siSmad2/3, sc-37238), siBAMBI (Origene) or 5 pmol of non-targeting siRNA (siNC, sc-37007) using Lipofectamin® RNAiMAX Reagent, according to the instructions provided by the manufacturers. All siRNAs were obtained from Santa Cruz Biotechnologies.

### RNA isolation

RNA was isolated from cells as previously described^20^. Briefly, cells were washed with cold PBS and fixed in RNAlater (Qiagen). Total RNA was isolated using RNeasy kit (Qiagen, Valencia, CA), following the manufacturer’s protocol, and treated in-column with DNase I. RNA concentration and quality were measured using the Agilent 2100 Bioanalyzer.

### Microarrays analysis

Five micrograms of total RNA from human TM primary cultures with transient silenced autophagy and controls were independently hybridized to human Clariom D microarrays (ThermoFisher) following the manufacturer’s instructions at the Duke Microarray Core Facility. Data analysis was performed using the Partek Flow and Partek Genome Suite statistical analysis software (Partek Incorporated). For this, raw data from the six hybridizations were imported to Partek Flow for alignment and quality controls. Aligned reads were quantified to transcriptome, filtered out on low expression and normalized. Feature gene lists with differential gene expression were generated and exported to Partek Genome Suite for statistical analysis using ANOVA with multiple test correction. P value lower than 0.05 was established for significance. Integrated pathway enrichment and functional analyses of the set of genes differentially expressed with inhibited autophagy in human TM cells were conducted with MetaCore (Clarivate Analytics). Array data were deposited at the Gene Expression Omnibus (National Center for Biotechnology; Accession Number: GSE122652).

### Quantitative real-time PCR

qPCR was performed as described in previous studies^20^. cDNA was synthesized from total RNA (1 µg) using oligo(dT) primer and Superscript II reverse transcriptase (Invitrogen, Carlsbad, CA). Real-time PCRs were performed in a 20 µL mix reaction [1 µL of cDNA (1:5 dilution), 10 µL iQ SYBR Green Supermix (Bio-Rad, Hercules, CA), 500 nm of each primer], in the BIO-RAD iCycler iQ system (Bio-Rad, Hercules, CA). The following PCR parameters were used: 95 °C for 5 min, followed by 50 cycles of 95 °C for 15 s, 60 °C for 15 s, and 72 °C for 15 s. The fluorescence threshold value (Ct) was calculated using the iCycle iQ system software. The absence of nonspecific products was confirmed by analysis of the melt curves and electrophoresis in agarose gels. The average Ct value of the following housekeeping genes (β-Actin, GAPDH) served as internal standard of mRNA expression. The expression levels were calculated using the formula 2^−Ct^, where ΔCt = Ct_gene_−Ct _average housekeeping_. The sequences of the primers used for the amplifications are the following: TGFβ2 (F: ATTGCTGCCTACGTCCACTT, R: TAAGCTCAGGACCCTGCTGT), BAMBI (F:TGCTGCTCACCAAAGGTGAAAT, R:CATCGTGCAGCCCTCTGTAA), SMA (F:AGCCAAGCACTGTCAGGAATC R: TACAGAGCCCAGAGCCATTG), HMGA2 F:AGACCTAGGAAATGGCCACAA, R: CCTAGTCCTCTTCGGCAGAC), β-Actin (F: TCCCTGGAGAAGAGCTACGA, R: AGGAAGGAAGGCTGGAAGAG), GAPDH (F: TGTCCCCACCCCCAACGTGT, R: CCCTCGGACGCCTGCTTCAC)

### Protein whole cell lysate & western blots

Protein lysates were obtained from cells as described earlier with some modifications^8,19^. After two washes in cold PBS, cells were lysed in cold RIPA buffer containing protease and phosphatase inhibitor cocktails (Thermo Scientific). Lysates were subjected to three freeze/thaw cycles and clarified by centrifugation at 12,000 × g for 30 minutes at 4 °C. Protein concentration was quantified using Micro BCA kit (Thermo Scientific). Protein lysates (5–10 μg) were separated by polyacrylamide SDS-PAGE gels (7–15%) and transferred to PVDF membranes (Bio-Rad). Membranes were blocked with 5% nonfat dry milk in 0.1% Tween-20/TBS and incubated overnight with primary antibodies, followed by incubation with a secondary antibody conjugated to horseradish peroxidase. Bands were detected using a chemiluminescence substrate (ECL; GE Healthcare and ECL2, Thermo Scientific). The following antibodies and dilutions were used: Atg5 (D5F5U, Cell Signaling, 1:1000), Atg7 (NBP1-95872, Novus Biologicals, 1:2000), LC3 (PM036, MBL, 1:1000), p62 (P0067, Sigma, 1:5000), SMA (A2547, Sigma, 1:4000), FN1 (sc-8422, Santa Cruz Biotechnologies, 1:500), Smad2/3 (3102S, Cell Signaling, 1:1000), pSmad2/3 (8828S, Cell Signaling, 1:1000),, ColI (Rockland, 600-401-103-0.1, 1:5000), GAPDH (SC-20357, Santa Cruz Biotechnologies, 1:500), β-actin (sc-69879, Santa Cruz Biotechnologies, 1:1500). Blots were scanned, mounted on Adobe Photoshop and analyzed by densitometry with Image J. β-Actin or GAPDH levels for each blot were used for loading control. Unless otherwise indicated, quantitative comparisons were conducted on samples on the same gel. Each specific blot was re-probed without stripping three or four times based on MW of the evaluated proteins and species of secondary antibody to avoid overlapping and recognition of the bands. Membranes were rinsed in 0.1% Tween-20/TBS and re-blocked blocked with 5% nonfat dry milk in 0.1% Tween-20/TBS between the probes. Antibody specificity was assessed by MW of the band and inclusion of positive and negative controls, including specific siRNAs, when possible. Representative images of the different antibodies tested include blots from the same independent experiment. Full-length blots are included as Supplemental Material.

### ELISA

Secreted TGFβ2 in the culture media was quantified by ELISA (Invitrogen) following the instructions provided by the manufacturer. Briefly, culture media was clarified by centrifugation, and latent TGFβ2 activated to the immunoreactive form by incubation for ten minutes at room temperature with 1N HCl, then neutralized with 1.2N NaOH/0.5 M HEPES. TGFβ2 concentrations were normalized to total protein concentration in corresponding whole cell lysates.

### GFP-LC3 puncta assay

To monitor autophagy by live cell imaging, were transduced human TM cells with 5 pfu/cell of the replication-deficient adenovirus AdGFP-LC3 (kindly provided by Dr. Wen-Xing Ding, University of Kansas Medical Center). AdGFP was used as a control. Three days after transduction, the cell culture media was changed with TGFβ2 (10 ng/mL)-containing media and the cells were further incubated at 37 °C, 5% CO_2_ for 48 h. For starvation condition, the cell culture media was changed with Hank’s balanced salt solution (HBSS, GIBCO) and it was incubated for 6 h. Images were acquired with CELENA® S Digital Imaging System (Logos biosystems) and the images were processed by using Fiji, an image processing package.

### Electron microscopy

Thin sections were prepared as describe in earlier studies^8,19^. Cells were washed in PBS and fixed in 2.5% glutaraldehyde / 2% paraformaldehyde in 0.1 M cacodylate buffer (pH 7.2). Fixed cells were detached by gentle scraping, pelleted, postfixed in 1% osmium tetroxide in 0.1 M cacodylate buffer and processed for transmission electron microscopy in the Morphology Facility at Duke University Eye Center. Thin sections (65 nm) were examined by electron microscopy (JEM-1200EX; JEOL USA, Peabody, MA).

### Statistical analysis

Experiments were performed at least three times using different cell strains from different donors. Data are represented as mean ± SD. Statistical significance was calculated using Student’s t-test (*) for two-group comparisons, one-way ANOVA with multiple comparisons (†) for more than two-group comparisons, or two-way ANOVA with Bonferroni (‡) for multiple variables (Prism; GraphPad, San Diego, CA). p < 0.05 was considered statistically significant. Mean values ± SD and P values have been included either in text or, when indicated, in Supplemental Material.

## Results

### Differential gene expression profile of autophagy-deficient TM cells

**Figure 1:**
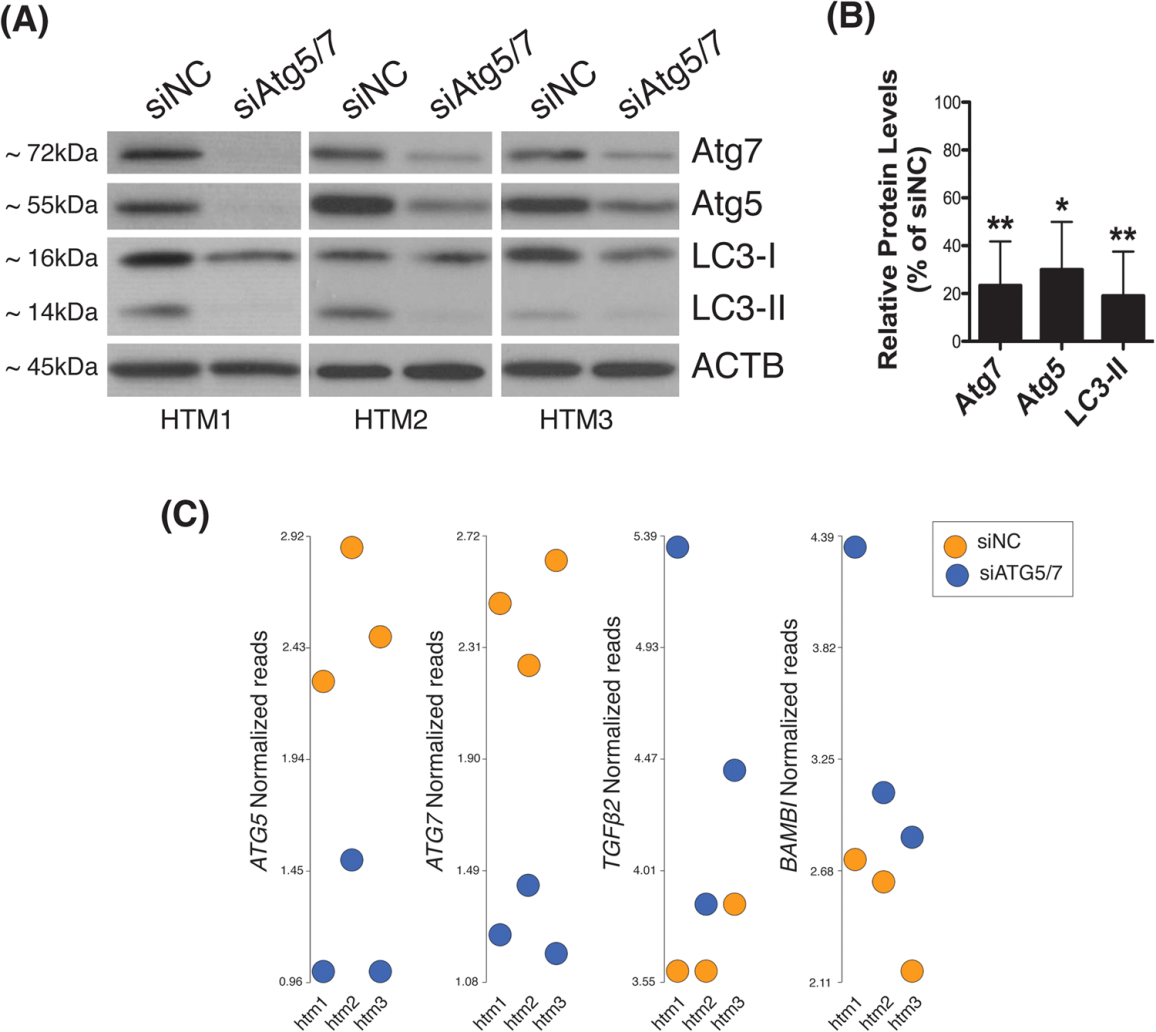
Autophagy inhibition in siAtg5/7-transfected TM cells. Three independent strains of primary human primary TM cells were transfected for 72 h with a cocktail of siRNAs targeting Atg5 and Atg7 (siAtg5/7). A scrambled siRNA (siNC) was used as control. (**A**) Protein levels of Atg5, Atg7, and LC3-II in all the three cell strains evaluated by western blot at 72 h post-transfection in whole cell lysates (5 μg). (**B**) Relative protein levels quantification as calculated from densitometric analysis of the bands, expressed as percentage of siNC controls. β-actin was used as loading control. *p < 0.05, **p < 0.01, t-test, n = 3. (**C**) Partek Flow dot plot visualization of the normalized reads confirming the upregulated expression of TGFβ2 and BAMBI genes and the downregulation of Atg5 and Atg7 in all the three cell strains.

**Figure 2:**
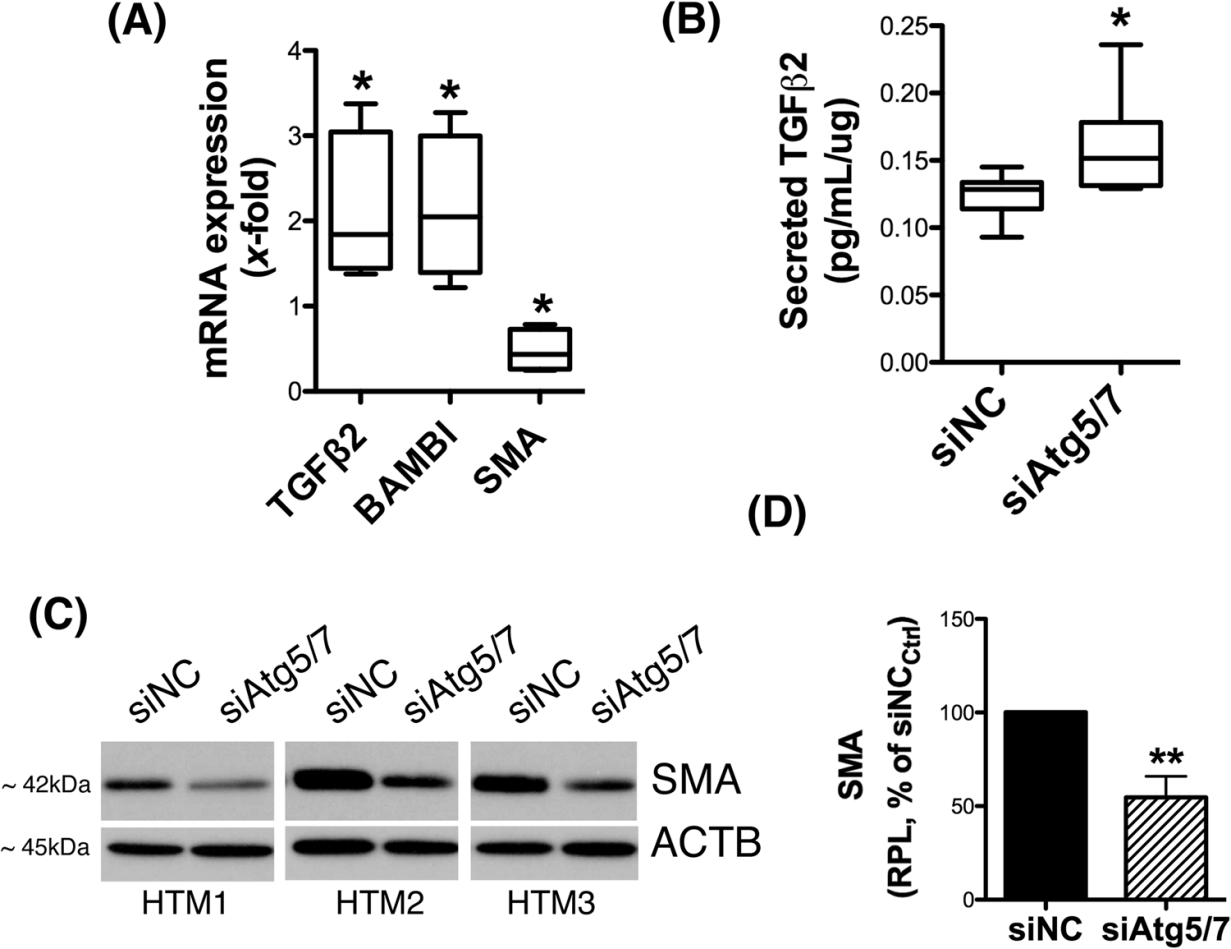
Decreased expression of fibrotic markers in siAtg5/7-transfected TM cells. (**A**) qPCR analysis confirming changes in expression levels of TGFβ2, BAMBI and SMA genes in siAtg5/7-transfected TM cells. (**B**) Levels of total TGFβ2 (latent and active) in culture media from siAtg5/7-transfected TM cells quantified by ELISA and normalized with total μg of corresponding whole cell lysates. (**C**) Protein levels of SMA as evaluated by western blot in whole-cell lysates (5 μg) from three independent human TM primary cells transfected for 72 h with siAtg5/7. (**D**) Mean SMA relative protein levels quantification as calculated from densitometric analysis of the bands, expressed as percentage of siNC controls. β-actin was used as loading control. *p < 0.05, **p < 0.01, t-test, n = 3. RPL: Relative protein levels.

In order to investigate the effects of downregulated autophagy in TM cells, we analyzed gene expression analysis in human TM cells deficient in autophagy. For this, three independent strains of primary human TM cells were transfected with a cocktail of siRNAs to specifically silence the expression of the autophagy genes Atg5 and Atg7 (siAtg5/7). A scrambled siRNA (siNC) was used as control. Downregulated protein levels of Atg5 (30.01 ± 19.97%, p = 0.02, n = 3) and Atg7 (23.33 ± 18.48%, p = 0.01, n = 3) in all the three cell strains were validated by WB at 72 h post-transfection (Fig. 1A,B). In addition, siAtg5/7-transfected cultures showed a significant decreased in the levels of the autophagosome marker LC3-II (19.12 ± 18.52%, p = 0.01, n = 3), thus confirming inhibition of autophagy (Fig. 1A,B). Changes in whole transcriptome profiling were evaluated by gene array analysis using Affymetrix Human Clariom D chips in conjunction with Partek Flow and Genomic Suite analysis software. Comparative analysis revealed 364 and 298 genes significantly up-regulated and down-regulated, respectively, more than 1.5-fold in autophagy-deficient TM cells compared to control. Tables 1 and 2 show the list of known genes significantly up- and down-regulated, respectively, more than 2-fold in siAtg5/7- compared to siNC-transfected cell. A complete list of genes with differential expression greater than 1.5 fold is included in Supplementary Dataset (Tables S1 and S2). Among them, we shall highlight the upregulated expression of TGFβ2 (2.04 fold, p = 0.02) and its pseudoreceptor BMP and activin membrane bound inhibitor (BAMBI) genes (2.17 fold, p = 0.008), as well as the downregulation of the fibrotic marker α-smooth muscle actin (SMA, −1.56 fold, p = 0.003, Table S2) in autophagy-deficient TM cells. Figure 1C shows the Partek Flow dot plot visualization of the normalized reads confirming the upregulated expression of TGFβ2 and BAMBI genes in all the three siAtg5/7-transfected cell strains, as well as the expected downregulation of Atg5 and Atg7. Quantitative PCR analysis in additional cell strains confirmed the observed upregulated expression of TGFβ2 (2.11 ± 0.88 fold, p < 0.05, n = 3) and BAMBI (2.14 ± 0.85 fold, p < 0.05, n = 3), as well as the downregulation of SMA (0.475 ± 0.24 fold, p < 0.05, n = 3, Supplementary Dataset - Table S2) in siAtg5/7-transfected cells (Fig. 2A). Increased total secreted TGFβ2 (0.16 ± 0.05 vs 0.12 ± 0.01 pg/mL/μg, p < 0.05, n = 5) and decreased SMA (54.57 ± 11.24%, p = 0.02, n = 3) protein levels in autophagy-deficient cells compared to siNC were additionally confirmed by ELISA (Fig. 2B) and WB (Fig. 2C,D), respectively. No significant changes in secreted TGFβ1 was observed (Supplemental Material-Fig. 2).

**Table 1:** List of known genes significantly up-regulated more than 2-fold in siAtg5/7- compared to siNC-transfected HTM cells.

GeneID	Description	Fold	P-Value
IFI44L	Interferon Induced Protein 44 Like	4.97	3E-03
IFIT1	Interferon Induced Protein With Tetratricopeptide Repeats 1	4.44	2E-04
DDX60	DExD/H-Box Helicase 60	3.48	6E-04
STX12	Syntaxin 12	3.09	3E-03
PLCE1-AS1	PLCE1 Antisense RNA 1	2.89	4E-02
USP41	Ubiquitin Specific Peptidase 41	2.88	1E-03
MX1	MX Dynamin Like GTPase 1	2.77	3E-03
SLC33A1	Solute Carrier Family 33 Member 1	2.68	6E-03
PAQR5	LIM domain only protein 3	2.68	5E-02
CDK14	Cyclin-dependent kinase 14	2.55	9E-04
RSAD2	Radical S-Adenosyl Methionine Domain Containing 2	2.48	4E-04
MESDC2	Mesoderm Development Candidate 2	2.47	1E-03
SULF1	Sulfatase 1	2.46	9E-04
OAS2	2′-5′-Oligoadenylate Synthetase 2	2.45	4E-02
SAMHD1	Deoxynucleoside triphosphate triphosphohydrolase	2.41	1E-03
LMO3	LIM domain only protein 3	2.39	9E-03
IFI44	Interferon Induced Protein 44	2.37	2E-04
ECM2	Extracellular matrix protein 2	2.35	3E-02
IFIT3	Interferon-induced protein with tetratricopeptide repeats 3	2.34	8E-04
FKBP14	FK506 Binding Protein 14	2.33	1E-03
SMOC1	SPARC-related modular calcium-binding protein 1	2.30	5E-04
DIO2	Type II iodothyronine deiodinase	2.27	1E-02
ATM	Serine-protein kinase ATM	2.18	2E-02
BAMBI	BMP and activin membrane-bound inhibitor homolog	2.17	8E-03
FGL2	Fibrinogen Like 2	2.16	3E-03
MAP2	Microtubule Associated Protein 2	2.15	4E-02
ISCA1P1	Iron-Sulfur Cluster Assembly 1 Pseudogene 1	2.14	2E-02
OAS1	2′-5′-oligoadenylate synthase 1	2.13	2E-02
TLR3	Toll-like receptor 3	2.12	5E-03
MICAL2	Microtubule Associated Monooxygenase, Calponin And LIM Domain Containing 2	2.10	5E-04
TGFB2	Transforming growth factor beta-2	2.04	2E-02
TSC22D1	TSC22 Domain Family Member 1	2.04	4E-04
RNA5SP355	RNA, 5S Ribosomal Pseudogene 355	2.02	3E-03
PHACTR2	Phosphatase And Actin Regulator 2	2.02	3E-04
ENDOD1	Endonuclease domain-containing 1 protein	2.01	2E-02
ZKSCAN1	Zinc finger protein with KRAB and SCAN domains 1	2.00	3E-03
HSPA4L	Heat shock 70 kDa protein 4 L	2.00	4E-02

**Table 2:** List of known genes significantly down-regulated more than 2-fold in non-stretched.

GeneID	Description	Fold	P-Value
RN7SL303P	RNA, 7SL, cytoplasmic 303, pseudogene	−17.42	4.6E-02
EIF2B5-AS1	EIF2B5 antisense RNA 1	−11.34	3.8E-02
TMEM238	transmembrane protein 238	−3.31	3.7E-02
PLAC9	placenta specific 9	−3.24	4.0E-02
MTND4P23	mitochondrially encoded NADH:ubiquinone oxidoreductase core subunit 4 pseudogene 23	−3.01	1.6E-02
ATG5	Autophagy Related 5	−2.69	4.6E-05
MTND5P2	mitochondrially encoded NADH:ubiquinone oxidoreductase core subunit 5 pseudogene 2	−2.64	4.3E-03
KRTAP2-3	keratin associated protein 2–3	−2.59	3.7E-03
MTND4P21	mitochondrially encoded NADH:ubiquinone oxidoreductase core subunit 4 pseudogene 21	−2.54	1.7E-03
AGR2	anterior gradient 2, protein disulphide isomerase family member	−2.42	2.4E-03
MTCO1P28	mitochondrially encoded cytochrome c oxidase I pseudogene 28	−2.38	1.3E-02
SCAANT1	SCA7/ATXN7 antisense RNA 1	−2.38	1.3E-02
AC138744.2	ATP synthase, H+ transporting, mitochondrial Fo complex subunit F2 pseudogene 4	−2.36	3.3E-02
MTND5P4	mitochondrially encoded NADH:ubiquinone oxidoreductase core subunit 5 pseudogene 4	−2.36	2.5E-02
CFL1P7	cofilin 1 pseudogene 7	−2.34	1.7E-03
PROSER3	proline and serine rich 3	−2.31	2.6E-02
MTATP6P25	mitochondrially encoded ATP synthase 6 pseudogene 25	−2.19	4.0E-02
SNRPCP18	small nuclear ribonucleoprotein polypeptide C pseudogene 18	−2.17	1.4E-02
HOXB7	homeobox B7	−2.17	2.8E-02
HMGA2	high mobility group AT-hook 2	−2.16	3.3E-04
SPX	Spexin Hormone	−2.16	1.5E-02
FRMPD1	FERM And PDZ Domain Containing 1	−2.16	2.7E-02
MTCYBP13	Mitochondrially Encoded Cytochrome B Pseudogene 13	−2.16	4.2E-02
RN7SKP187	RNA, 7SK Small Nuclear Pseudogene 187	−2.09	2.9E-02
RPS24P7	Ribosomal Protein S24 Pseudogene 7	−2.07	9.8E-03
DLX5	Distal-Less Homeobox 5	−2.07	4.2E-02
HBA2	Hemoglobin Subunit Alpha 2	−2.05	2.8E-02
ACOT4	Acyl-CoA Thioesterase 4	−2.05	4.2E-03
MIR4731	MicroRNA 4731	−2.02	5.0E-03
MTRNR2L3	Humanin-Like Protein 3	−2.01	1.6E-02
MTND4P9	Mitochondrially Encoded NADH:Ubiquinone Oxidoreductase Core Subunit 4 Pseudogene 9	−2.01	2.7E-02
SUMO2P13	SUMO2 Pseudogene 13	−2.00	3.9E-02

### Functional network analysis of gene expression changes in autophagy-deficient TM cells

In order to identify pathways and regulatory elements associated with observed changes in gene expression in autophagy-deficient human TM cells, we further evaluated the gene lists obtained from microarray analysis using MetaCore, a pathway analysis and data mining software. Table 3 shows the top pathways and networks identified in siAtg5/7-transfected human TM cells. Genes with differential gene expression associated to these pathways are listed. As seen, the interferon signaling pathway, angiotensin system, and regulation of endothelial-mesenchymal transition (EMT) were the pathways more significantly enriched in human TM cells with silenced Atg5/Atg7 expression.

**Table 3:** List of the objects from metacore enrichment analysis, pathway maps.

Maps	p-value	Network objects from data
Protein folding and maturation_Angiotensin system maturation \ Human version	4.289E-07	Angiotensinogen, Angiotensin (IV, I, II, III)
Immune response_Antiviral actions of Interferons	2.177E-05	STAT1, OAS1, STAT2, MxA, RNaseL, OAS2
DNA damage_Brca1 as a transcription regulator	1.206E-04	STAT1, Cyclin D1, ATF-1, XPC, ATM
Development_Angiotensin signaling via STATs	1.660E-04	STAT1, STAT2, Calcineurin A, IP3 receptor, Angiotensin II
Development_TGF-beta-dependent induction of EMT via SMADs	2.570E-04	TGF-beta 2, TGF-beta, HMGA2, ACTA2, N-cadherin, PAI1
Immune response_Oncostatin M signaling via JAK-Stat	4.237E-04	STAT1, Cyclin D1, ACTA2, LIFR
Development_BMP7 in brown adipocyte differentiation	4.321E-04	DIO2, EDNRA, Angiotensinogen, Cytochrome c, ENDOD1
Immune response_IFN alpha/beta signaling pathway	5.997E-04	STAT1, STAT1/STAT2, ISG54, STAT2
Development_Regulation of epithelial-to-mesenchymal transition (EMT)	6.453E-04	TGF-beta 2, ACTA2, EDNRA, N-cadherin, PAI1, PDGF-D

### Autophagy modulates TGFβ-induced fibrogenesis in TM cells

**Figure 3:**
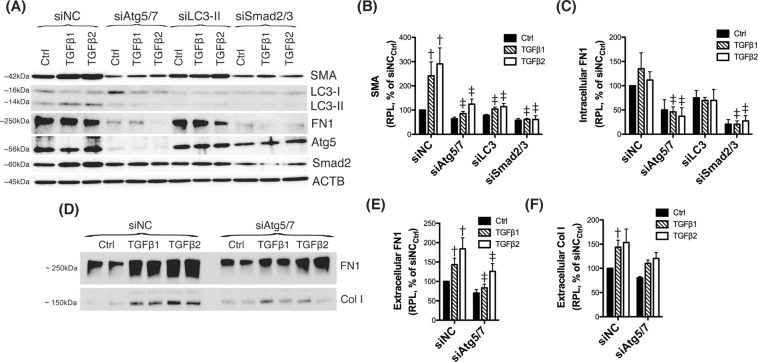
Silencing autophagy genes inhibits TGFβ-induced fibrosis. Three independent strains of human TM primary cells were transfected with siRNAs targeting Atg5/Atg7 (siAtg5/7), LC3 (siLC3), or Smad2/3 (siSmad2/3). A scrambled siRNA (siNC) was used as control. At 2 d.p.t, cells were treated with either TGFβ1 or TGFβ2 (10 ng/mL) for 48 h. (**A**) Protein levels of SMA, FN1, Atg5, LC3, and Smad2 evaluated by western blot in whole cell lysates (5 μg). (**B**) Relative protein levels quantification of the fibrotic markers SMA (**B**) and FN1 (**C**) calculated from densitometric analysis of the bands, expressed as percentage of siNC_Ctrl_ (set to 100%). β-actin was used as loading control. (**D**) Protein levels of FN1 and Col I in conditioned-culture media (20 μL) analyzed by western-blot. (**E,F**) Specific bands were quantified by densitometry and normalized with total protein (μg) of corresponding whole cell lysates, expressed as percentage of siNC. ^†^Denotes statistical significance when comparing TGFβ treatment versus control, using one-way ANOVA with multiple comparisons; ^‡^denotes statistical significance when comparing TGFβ-treated siAtg5/7, siLC3 or siSmad2 versus their respective siNC treated control, using two-way ANOVA with Bonferroni post-test. Specific mean ± SD and p values, as well as densitometric analysis confirming significant knocked down expression of LC3, Atg5, and Smad2 are included in Supplemental Material. RPL: Relative protein levels.

EMT transition and fibrosis have been implicated in outflow pathway pathophysiology in glaucoma^21–23^; therefore, we decided to further investigate a potential role of autophagy in regulating this particular pathway. As seen earlier, knocking down Atg5 and Atg7 genes resulted in a decrease in the constitutive levels of the EMT/fibrotic marker SMA. Next, we investigated whether silencing of siAtg5/7 could also affect TGFβ-dependent induction of EMT. For this, human TM cells were transfected with siAtg5/7, as detailed earlier, and treated two days after transfection with TGFβ1 or TGFβ2 (10 ng/mL). Protein levels of SMA were evaluated by WB. Cells transfected with siSmad2/3 served as positive control for TGFβ signaling. For clarity, mean values ± SD and P values, as well as densitometric analysis confirming successful knocked down levels have been included in Supplemental Material. As expected, treatment with TGFβs induced the expression of SMA in siNC-transfected cells compared to control, which was blocked in siSmad2/3-deficient cells (Fig. 3A,B). Interestingly, downregulation of the autophagy genes Atg5 and 7 significantly reduced the TGFβ-dependent increase in SMA. To confirm whether this inhibitory effect was applicable to other EMT-related genes, we additionally looked at the intracellular and secreted protein levels of fibronectin (FN1) and collagen I (Col I). As shown in Fig. 3C–F, silencing Atg5 and Atg7 also decreased the constitutive and the TGFβ-induced expression of FN1 and COL I, although no statistical significance was reached in this later.

**Figure 4:**
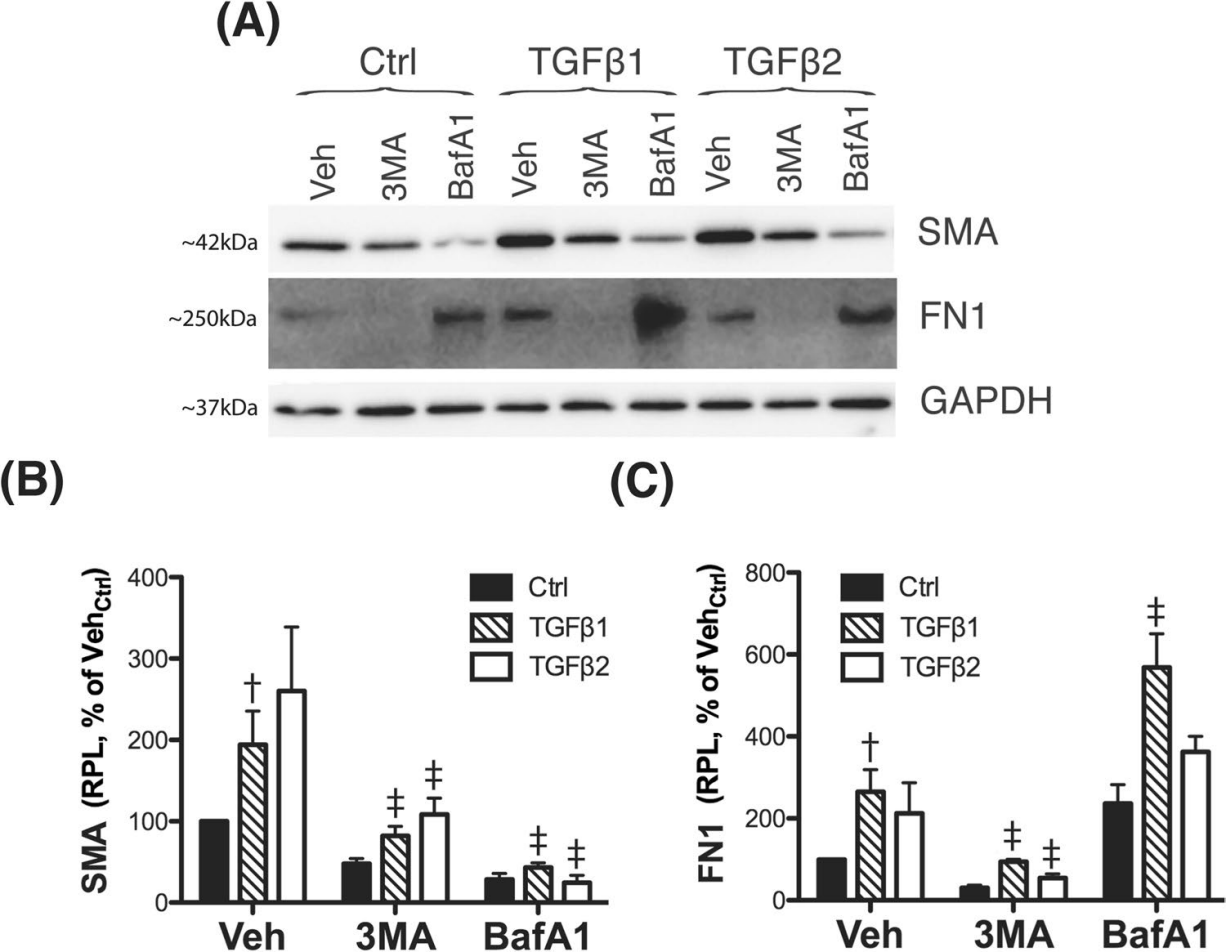
Pharmacological inhibition of autophagy regulates TGFβ-induced fibrosis. (**A**) Three independent strains of human TM primary cells were treated for 24 h with the autophagy inhibitors 3-MA (10 mM) or BafA1 (100 nM). Protein levels of SMA and FN1 were evaluated in whole-cell lysates (5 μg) by western-blot. SMA (**B**) and FN1 (**C**) relative protein quantification was calculated from densitometric analysis of the bands using GAPDH as loading control. Data is expressed as percentage of Veh_Ctrl_. ^†^Denotes statistical significance when comparing TGFβ treatment versus control, using one-way ANOVA with multiple comparisons; ^‡^denotes statistical significance when comparing effect of each autophagy inhibitor versus their respective TGFβ-treated control, using two-way ANOVA with Bonferroni post-test. Specific mean ± SD and p values are included in Supplemental Material. RPL: Relative protein levels.

Atg5 and Atg7 have been reported to fulfill other non-autophagic roles. This raised the question on whether the observed effect of siAtg5/7 on the expression of EMT-related genes was mediated by inhibition of autophagy or, in contrast, through a non-autophagy role of Atg5 or Atg7. To distinguish between these possibilities, we checked SMA and FN1 expression in siLC3-transfected cells (Fig. 3), as well as in TM cells treated with the autophagy inhibitors, 3-MA (10 mM) or BafA1 (100 nM) (Fig. 4). In all the three cases, blockage of the autophagy significantly lowered the constitutive and TGFβ-induced levels of SMA (Fig. 4B and Supplemental Material). siLC3 and more strongly 3-MA also diminished FN1 levels (Fig. 4C and Supplemental Material). Interestingly, blockage of lysosomal degradation by BafA1 resulted in higher FN1 content, suggesting that intracellular FN1 is targeted for lysosomal degradation.

### Upregulation of BAMBI in autophagy-deficient TM cells inhibits TGFβ/Smad signaling

**Figure 5:**
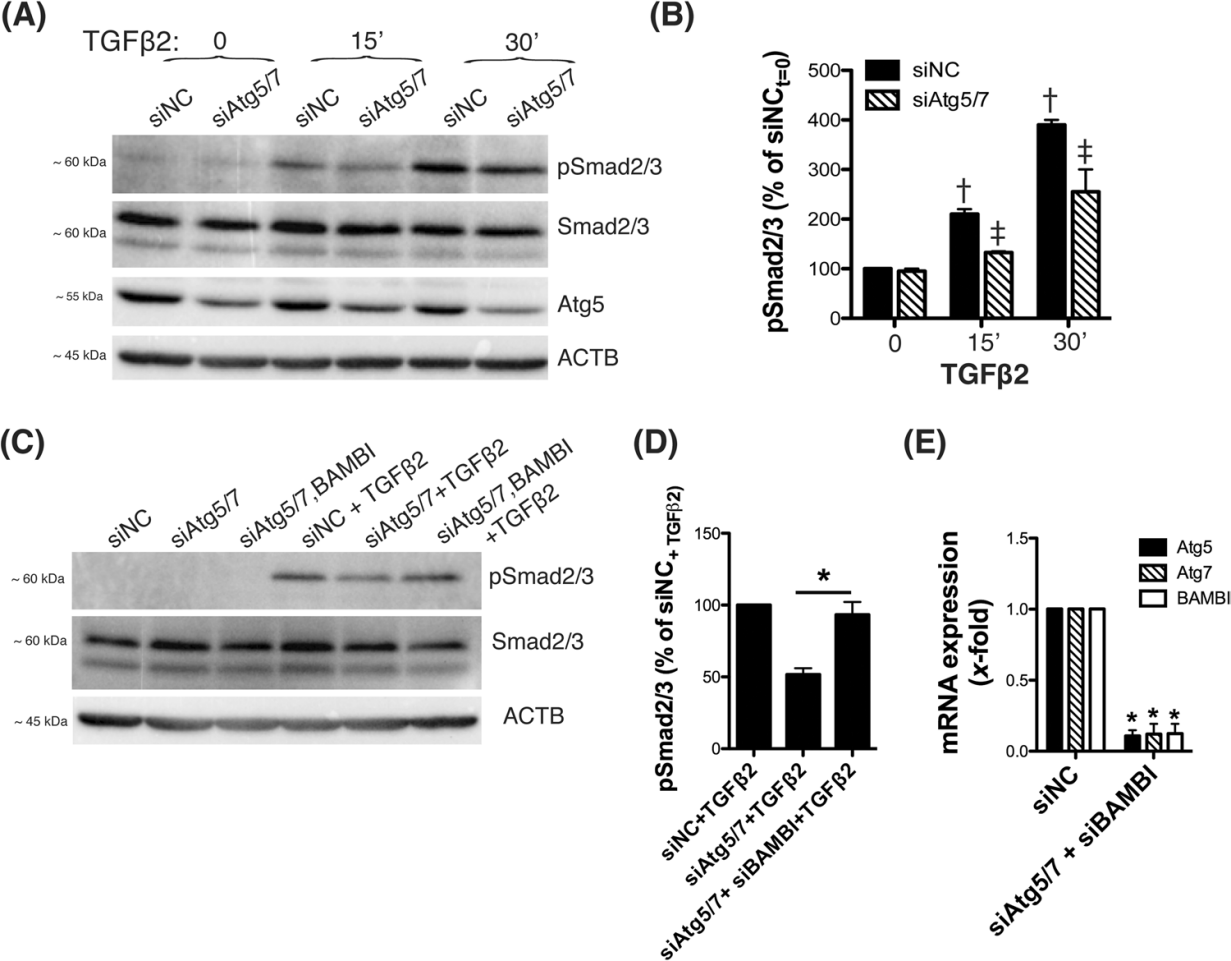
BAMBI-mediated reduced activation of Smad2/3 signaling in autophagy-deficient cells. Three independent strains of human TM primary cells were transfected with siAtg5/7 or siNC as control. At 2 d.p.t, cells were treated with TGFβ2 (10 ng/mL). (**A**) Phosphorylated and total Smad2/3 protein levels were evaluated by western blot in whole cell lysates (5 μg). (**B**) Relative pSmad2/3 levels compared to total Smad2/3 were calculated from densitometric analysis of the bands and expressed as percentage of siNC at t = 0 post TGFβ2-treatment. ^†^Denotes statistical significance when comparing to control, using one-way ANOVA with multiple comparisons; ^‡^denotes statistical significance when comparing effect of siAtg5/7 at each time point, using two-way ANOVA with Bonferroni post-test. (**C**) Three independent strains of human TM primary cells were transfected with siAtg5/7 or siAtg5/7 + siBAMBI; siNC was used as control. At 2 d.p.t, cells were treated with TGFβ2 (10 ng/mL); pSmad2/3 and Smad2/3 protein levels evaluated by WB. (**D**) Relative pSmad2/3 levels post TGFβ2-treatment in siAtg5/7 and siAtg5/7/BAMBI-transfected cells were calculated from densitometric analysis of the bands and expressed as percentage of treated siNC. (**E**) qPCR analysis confirming downregulated expression of Atg5, Atg7 and BAMBI in transfected cells. In (**D,E**), *denotes statistical significance when comparing to control, using t-test. Specific mean ± SD and p values, as well as densitometric analysis confirming significant knocked down expression of Atg5 (**A**) and non-significant changes in the expression of Smad2 are included in Supplemental Material.

Next, we investigated activation of TGFβ signaling in autophagy-deficient TM cells by monitoring phosphorylation of Smad2/3 in response to TGFβ2 (10 ng/mL). As seen in Fig. 5A,B and Supplemental Material 2, siAtg5/7-transfected cells displayed lower levels of pSmad2/3 upon TGFβ2 treatment compared to control cells, indicating deficient activation of the canonical TGFβ/Smad signaling pathway. Microarray and qPCR analysis showed upregulated expression of BAMBI, an antagonist of TGFβ signaling^24^, with downregulation of Atg5/7 (Table 2, Fig. 2A). We tested whether such upregulation of BAMBI could mediate inhibition of TGFβ/Smad signaling. For this human TM cells were transfected with siAtg5/7 together with siBAMBI for 48 h and then treated with TGFβ2 (10 ng/mL) for 15 min. Interestingly, knocking down BAMBI expression almost enterally restored Smad2/3 phosphorylation in response to TGFβ2 treatment in autophagy-deficient TM cells (93.3 ± 15.27%, n = 3, p < 0.01, t-test, Fig. 5C,D). No differences in the levels of total Smad2/3 were observed with downregulation of Atg5/7 and BAMBI. Decrease Atg5 (0.1 ± 0.04 fold, n = 3, p < 0.0001) Atg7 (0.12 ± 0.07 fold, n = 3, p < 0.0001) and BAMBI (0.123 ± 0.068 fold, n = 3, p < 0.0001) mRNA levels in the transfected cells were confirmed by qPCR (Fig. 5E). Similarly, no significant changes in Smad2/3 phosphorylation was noted when transfecting siBAMBI in control cells (Supplemental Material). Unfortunately, BAMBI protein levels could not be confirmed since none of the three antibodies tested in our laboratory were capable of recognizing a band of the appropriate molecular weight of human BAMBI when validated with siBAMBI (data not shown).

### TGFβ1 and TGFβ2 dysregulate autophagy in a dose-dependent manner

**Figure 6:**
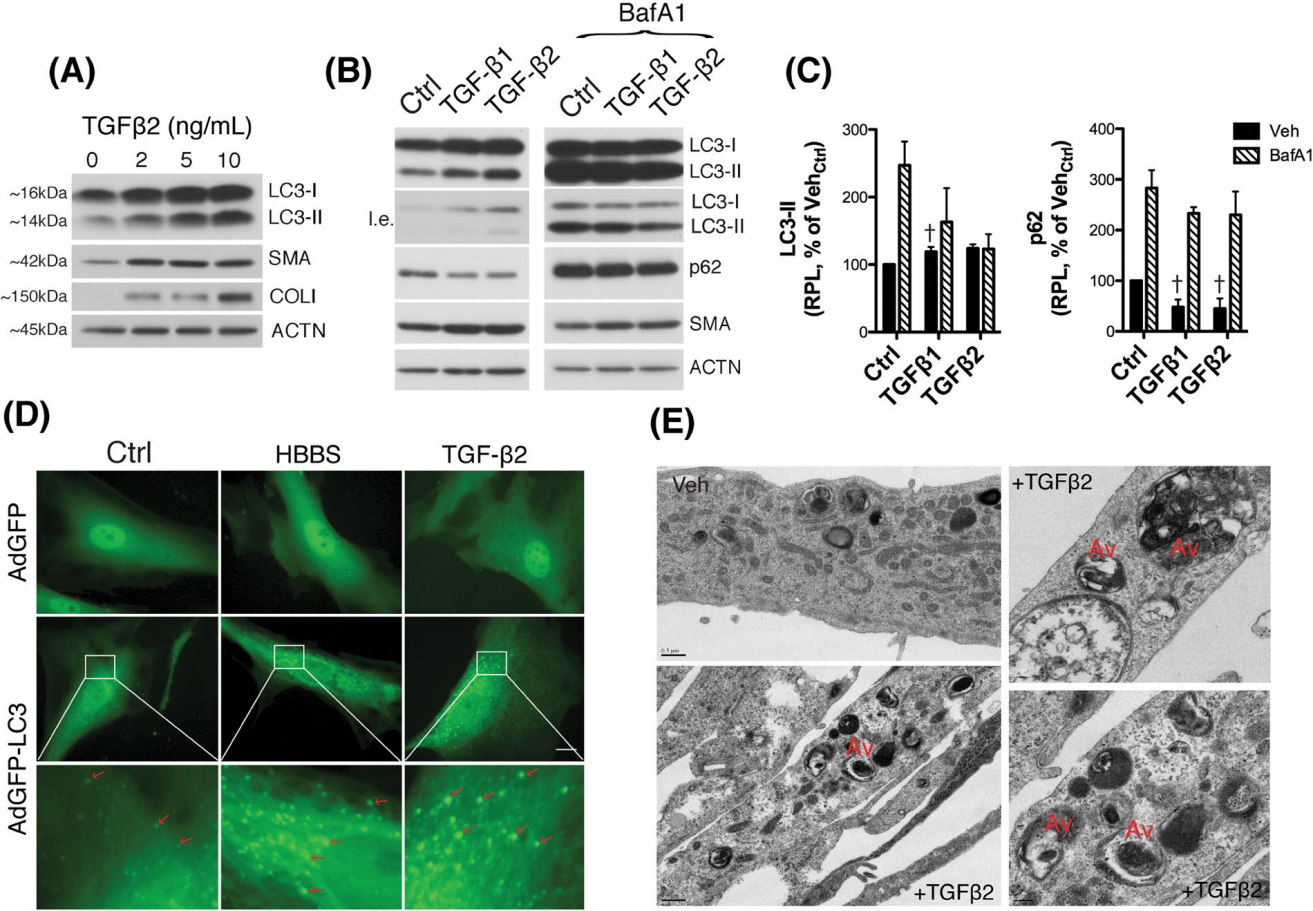
TGFβ treatment dysregulates autophagy in TM cells. (**A**) Human TM cells were treated with increasing concentrations of TGFβ2 (0, 2, 5, 10 ng/mL) for 48 h. Levels of LC3, SMA and Col I were evaluated in whole cell lysates (5 μg) by western blot. (**B**) Protein levels of LC3, p62 and SMA evaluated by western blot in TM cells treated with TGFβ1 or TGFβ2 (10 ng/mL) for 48 h in the absence or presence of BafA1 (100 nM) added to the culture media 24 h post-TGFβ treatment. (**C**) Relative quantification of p62 and LC3-II levels calculated from densitometric analysis of the bands, expressed as percentage of Veh_Ctrl_. Control and BafA1-treated samples derive from the same experiment and gels/blots were processed in parallel. β-actin was used as loading control. (**D**) TM cells transduced with either AdGFP or AdGFP-LC3 (5 pfu/cell) were treated with TGFβ2 (10 ng/mL). The presence of GFP-LC3 puncta (red arrows) was evaluated at 48 h using the CELENA® S Digital Imaging System. TM cells undergoing starvation by culturing in HBSS media were used as autophagy control. (**E**) Electron micrographs of TM cells treated with TGFβ2 (10 ng/mL) for 48 h showing the presence of autophagic vacuoles (Av). ^†^Denotes statistical significance using one-way ANOVA with multiple comparisons. Specific mean ± SD and p values, as well as densitometric analysis of SMA are included in Supplemental Material. RPL: Relative protein levels; l.e.: lower exposure.

LC3 immunoblot in Fig. 3A showed increased LC3-II in TGFβ1 and TGFβ2-treated cells. We wanted to follow up on this interesting observation. For this, we treated human TM cells with increasing concentrations of TGFβ2 (0, 2, 5, and 10 ng/mL) for 48 h and the levels of LC3-II, as well as of the positive controls SMA and COL I evaluated. As seen in Fig. 6A, TGFβ treatment triggered a dose-dependent increase in LC3-II levels. To discern whether such an increase in LC3-II was the result of autophagy activation or, in contrast, reduced flux, TGFβ treatment was conducted in the absence or presence of BafA1 (100 nM) and levels of p62, an autophagic receptor that is commonly used to monitor autophagic flux, were investigated in addition to those of LC3-II (Fig. 6B,C)^25^. Confirming our previous result, treatment with TGFβ1 and TGFβ2 (10 ng/mL) elevated LC3-II levels compared to non-treated cultures (Fig. 6C and Supplemental Material 2). A decrease in p62 was also observed. As expected, BafA1-treated cells demonstrated increased LC3-II and p62, compared to non-treated ones. However, no further increase, but rather a slight decrease, was observed in combination with TGFβ suggesting potential inhibition of autophagic flux. We further confirmed increased autophagosome number with TGFβ in GFP-LC3 transfected cells as well as in electron micrographs. See elevated number of green-puncta in TGFβ-treated cells, comparable to those observed in cells undergoing starvation (HBSS) not observed with AdGFP control [Fig. 6D, Supplemental Video (t = 10–17 h)], as well as the enhanced presence of autophagic vacuoles in electron micrographs (Fig. 6E).

### Smad2/3 mediates the dysregulation of autophagy by TGFβ

**Figure 7:**
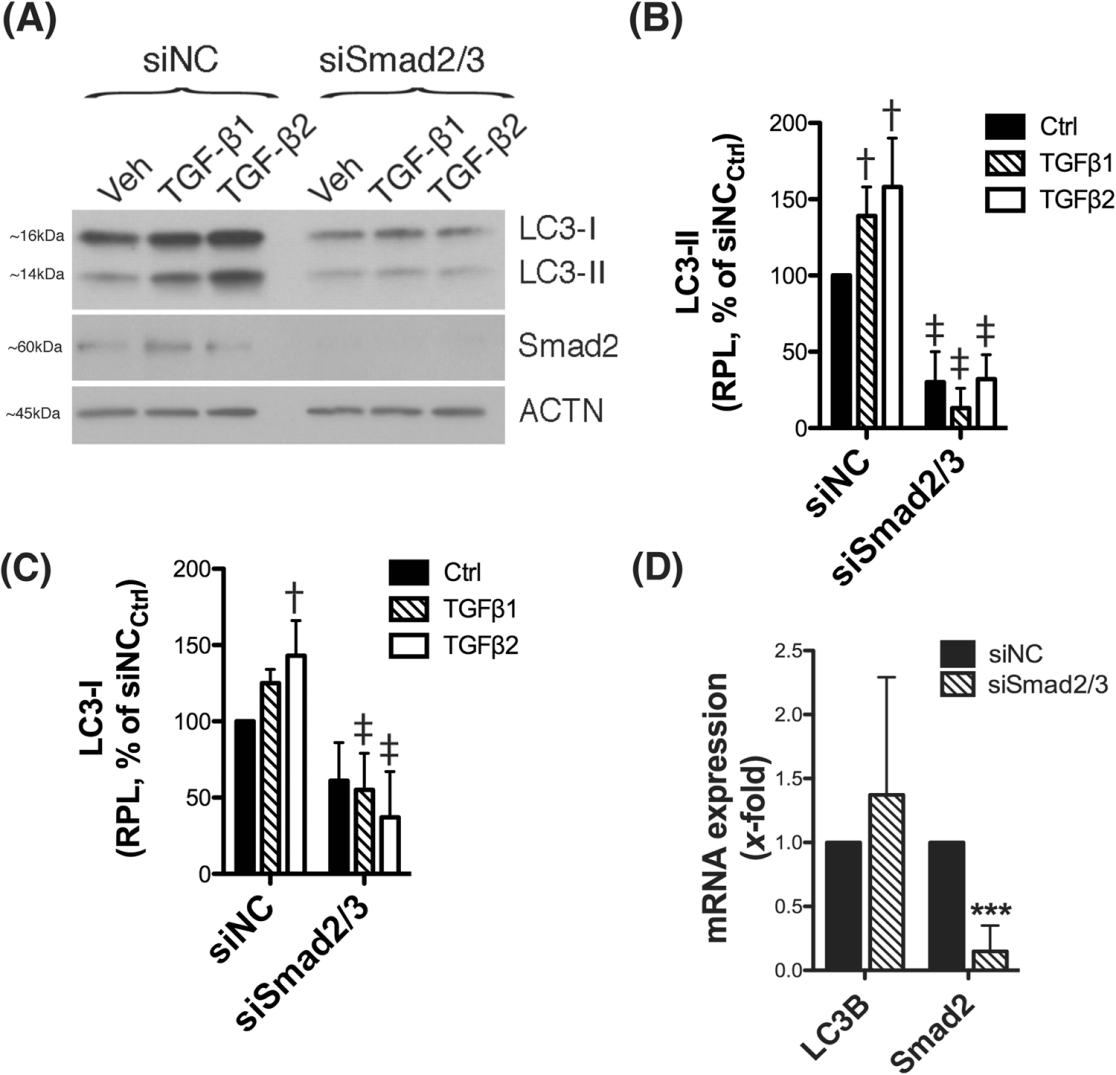
Smad2/3 mediates the dysregulation of autophagy by TGFβ in TM cells. Three independent strains of human TM primary cells were transfected for 72 h with siSmad2/3. A scrambled siRNA (siNC) was used as control. At 2 d.p.t, cells were treated with either TGFβ1 or TGFβ2 (10 ng/mL) for 48 h. (**A**) Protein levels of LC3 and Smad2 were evaluated by western blot in whole cell lysates (5 μg). (**B**,**C**) Relative protein levels quantification were calculated from densitometric analysis of the bands and expressed as percentage of siNC_Ctrl_. β-actin was used as loading control. (**D**) mRNA LC3B and Smad2 fold expression levels quantified by qPCR analysis. ^†^Denotes statistical significance when comparing TGFβ treatment versus control, using one-way ANOVA with multiple comparisons; ^‡^denotes statistical significance when comparing TGFβ-treated siSmad2 versus the respective siNC treated control, using two-way ANOVA with Bonferroni post-test. *Denotes statistical significance compared to siNC control using t-test. Specific mean ± SD and p values, as well as densitometric analysis confirming knocked down of Smad2/3 are included in Supplemental Material. RPL: Relative protein levels.

We next investigated whether the Smad2/3 canonical pathway is involved in the TGFβ-triggered dysregulation of autophagy in human TM cells. For this, we transiently silenced Smad2/3 expression via siRNA and the level of LC3 was analyzed. As seen in Fig. 7A and quantified in Fig. 7B,C and Supplemental Material, downregulation of Smad2/3 significantly decreased the constitutive and TGFβ-induced increased levels of LC3-II and those of LC3-I. Not significant changes at the messenger level of LC3 was observed with downregulated expression of Smad2/3 (Fig. 7D).

## Discussion

Here, we report for the first time (i) abrogation of TGFβ/Smad signaling in human TM cells deficient in autophagy, mediated by upregulation of BAMBI, (ii) a role of autophagy in regulating TGFβ-induced fibrogenesis, and (iii) the regulation of autophagy by TGFβ in TM cells. Also, ours represents the first study in the literature investigating the comparative gene expression profile of TM cells with silenced autophagy.

Silencing of autophagy was achieved by knock down of Atg5 and Atg7 genes, critical molecules for the induction of autophagy, participating in both conjugation cascades leading to LC3 lipidation and autophagosome formation^10^. The choice of silencing both genes together rather than individually came from empirical studies in our laboratory, in which we observed a much higher autophagy inhibition efficiency, close to 100% – as measured by monitoring LC3-II levels - when siRNA targeting those two genes were transfected together or with siLC3. Inhibition of autophagy in TM primary cells lead to significant changes in the transcriptome profile with >600 transcripts showing differential gene expression. Functional network analysis of these genes identified three major signaling pathways affected in Atg5/7-deficient TM cells: interferon signaling pathway, angiotensin system, and regulation of TGFβ-induced EMT. A role of autophagy or autophagy genes in these pathways is supported in the literature. For instance, Atg5 is known to act as a suppressor of innate antiviral immune signaling^26,27^, therefore it is not surprising to find interferon signaling as the more significant pathway altered in siAtg5/7-transfected cells. A priori, interferon system has not been reported to play a relevant role in outflow pathway pathophysiology, although it cannot be discarded that dysregulation of autophagy could compromise the immune privilege in the anterior chamber of the eye or predispose the trabecular meshwork to pathogen invasion and development of some secondary forms of glaucoma, such as uveitis glaucoma.

More relevant for outflow pathway physiology are the renin-angiotensin system and the EMT pathways. Members of the renin-angiotensin system, including angiotensin-converting enzyme (ACE), angiotensin II and AT1 have been detected in the AH and outflow pathway tissues, and are believed to play a critical role in regulating AH homeostasis^28^. ACE inhibitors have shown efficacy in lowering IOP in animal studies^29,30^ and in human patients with ocular hypertension and glaucoma^31^. The mechanisms and the exact site of action have not been elucidated. Stimulation of prostaglandin synthesis, ECM remodeling and/or fibrogenesis have been proposed^28^.

The role of TGFβ-induced fibrosis and EMT in the pathogenesis of ocular hypertension and glaucoma has been best characterized^21–23^. Elevated levels of bioactive TGFβ2 are present in the AH from glaucoma patients and are believed to contribute to the increased ECM deposition and fibrosis in the glaucomatous TM^4–6^. Moreover, perfusion of recombinant TGFβ2 in human anterior eye segments or adenoviral gene transfer of constitutively active TGFβ2 in mice and rats elevates IOP and reduces outflow facility^32^. TGFβ2 has been shown to induce TM cell transdifferentiation, as indicated by the expression of the myofibroblast marker SMA and several ECM-related genes (FN1, collagens)^21–23^. Intriguingly, SMA expression is lost with age and in POAG *in vivo* despite the presence of active TGFβ2 in the AH^32^. Our data here shows the upregulation and increased secretion of TGFβ2 in TM cells with silenced Atg5 and Atg7. Very interestingly, similar to the reports in the aging and POAG TM, SMA was found to be downregulated in Atg5/7-deficient cells even though the increase in TGFβ2 content. Furthermore, the induction of SMA in response to exogenous TGFβ1 or TGFβ2 treatment was dramatically reduced in siAtg5/7-transfected cultures. This effect was not restricted to SMA, but we also observed a decrease in the constitutive and TGFβ-induced levels of the fibrotic markers FN1 and Col I.

Since autophagy genes are known to play additional non-autophagic roles^33^, we considered the possibility that the observed inhibitory effect could be independent of autophagy. However, silencing LC3 or pharmacological inhibition of autophagy with 3-MA, which blocks autophagosome formation, or BafA1, which blocks autophagic degradation, also diminished constitutive and TGFβ-induced expression of SMA. Interestingly, these two drugs showed a differential effect on FN1 expression. While 3-MA decreased FN1 protein levels, BafA1 treatment increased them. A plausible explanation for this apparent contradicting data is that FN1 is intracellularly degraded within the lysosomes by autophagy; therefore, its levels increases when it can be transported to, but not degraded within the lysosomes. Altogether, the data strongly indicates a role of autophagy in regulating fibrogenesis in TM cells.

An interplay between autophagy and the fibrotic response is recently gaining attention in the literature. Intriguingly, depending on the cell type, tissue or the pathological settings, autophagy can positively or negatively regulate fibrosis. Atg7 knockdown and Atg5 knockout decreased the fibrotic effect of TGFβ in human atrial and mouse embryonic fibroblasts, respectively^34^. Inhibition of autophagy also repressed fibroblast to myofibroblast phenoconversion of primary cardiac fibroblasts^35^. Similarly, autophagy promoted profibrotic effects in hepatic stellate cells and human lung fibroblasts contributing to liver and idiophatic pulmonary fibrosis^36,37^. Blockage of autophagy by pharmacological and genetic approaches suppressed renal interstitial fibrosis in an *in vivo* mouse model of unilateral ureteral obstruction^38^. In contrast, silencing Atg5 in the liver during preneoplastic stage facilitated liver fibrosis and tumorigenesis^39^. In a very robust study, Newman *et al*. conducted global gene expression analysis in adenocarcinoma lung cells with downregulated Atg5 gene expression. Similar to our data, they found the majority of the differentially expressed transcripts to be either direct or indirect targets of transcriptional upregulation by TGFβ, including SMA^40^. However, in their case, autophagy was repressing the transcriptional activation by the TGFβ gene regulatory pathway, through TRAF3/RELB-mediated transcriptional repression of Smad proteins.

The regulation of cell signaling by autophagy or “signalphagy”^41^ is still an emerging field. Silencing Atg5/7 in TM cells had the same effect as silencing Smad2/3, suggesting that autophagy might regulate fibrosis by abrogating TGFβ/Smad signaling through the selective degradation of a negative regulator. Supporting this, phosphorylation of Smad2/3 in response to TGFβ2 treatment was inhibited in autophagy-deficient TM cells. Intriguingly, BAMBI, an antagonist of TGFβ signaling and inhibitor of BMP signaling^24^, was also found to be significantly upregulated with silenced Atg5 and 7. BAMBI is a transmembrane glycoprotein related to the TGFβ-family type I receptors that lacks the intracellular kinase domain. Overexpression of BAMBI suppresses the effect of TGFβ. More interestingly, BAMBI has been shown to be predominantly degraded through the autophagy lysosomal pathway^42^. BAMBI can negatively regulate TGFβ signaling through different mechanisms. In the cytosol, BAMBI can directly bind to TGFβ-receptors and abrogate Smad2/Smad3 signaling, BAMBI can also form a ternary complex with Smad7/ALK5/ TGFβRI and block Smad3 activation. In association with Smad2/3, BAMBI can translocate into the nucleus and modulate TGFβ-induced transactivation^24^. At the same time, TGFβ can regulate BAMBI transcription by direct binding of Smad3/4 to BAMBI promoter^43^, although a recent report has shown the downregulation of BAMBI following TGFβ2 treatment in TM cells^44^. BAMBI knockdown alters TM ECM expression and reduces outflow facility in mice, being BAMBI proposed as an important regulator of IOP homeostasis^45^, potentially beneficial in glaucoma. Very excitedly, silencing BAMBI expression in siAtg5/7-tranfected cells completely reestablished the levels of pSmad2 in response to TGFβ2 treatment. It is therefore possible that the upregulation of BAMBI in autophagy deficient TM cells could regulate the constitutive and TGFβ-induced expression of the fibrotic markers in Atg5/7-deficient TM cells. We are currently developing appropriate tools to further pursue this hypothesis.

Another novel finding here is the Smad2/3-mediated dysregulation of autophagy by TGFβ in TM cells. TGFβ treatment induced a dose-dependent increase in the levels of the autophagosome marker LC3-II, which was completely prevented with silenced Smad2/3 expression. Protein levels of LC3-I were also observed significantly decreased with abolished canonical TGFβ signaling, which pointed towards a potential Smad2/3-regulated transcription of LC3 gene. However, qPCR analysis was not conclusive among the different TM cell strains. An alternative explanation is Smad2/3 to affect the transcription of other Atg genes participant of the pro-LC3 to LC3-I or LC3-I to LC3-II conversions. The increase in LC3-II levels was accompanied by higher number of GFP-LC3 puncta and the presence of autophagic vacuoles, as observed by electron microscopy, in the TGFβ-treated cultures. Although all together indicates activation of autophagy, the decrease in p62 and the fact that no further increase in LC3-II levels were observed in the presence of BafA1 also suggest diminished autophagic flux. Both events do not exclude each other and can occur simultaneously^25^. Modulation of autophagy by TGFβ has been described in other cell types, but the effect seems to be highly cell type- and/or cell context- specific. TGFβ treatment activates autophagy in hepatocellular and mammary carcinoma cell lines^46^, renal tubular epithelial cells^47^, glomerular mesanglial cells and glioma cells^48^. In contrast, TGFβ treatment inhibited autophagy flux during myofibroblast differentiation in lung fibroblasts^49,50^. As mentioned earlier, numerous studies have confirmed elevated levels of TGFβ2 in the AH of patients with glaucoma. Based on our findings, such increased levels of TGFβ2 can be one of the triggers or contributing factors leading to the previously reported dysregulation of autophagy in glaucomatous TM cells. At the same time, as seen in autophagy-deficient TM cells, dysregulated autophagy can contribute to the upregulated expression of TGFβ2.

In summary, our data here shows for the first time an intricate interplay between autophagy and TGFβ signaling, and a role of autophagy in regulating fibrogenesis via BAMBI and Smad2/3 signalings in TM cells. The implication of autophagy in the induction of the fibrotic response opens a novel area for investigation of therapeutic targets for amelioration of fibrosis in the TM. Future studies will be directed at identifying the exact mechanisms regulating such crosstalk.

## Supplementary information

Supplementary Dataset

Live-cell imaging on autophagy induction in hTM cells in response to TGFβ2 treatment

Supplemental Material
