# Supplementary material for: Transcriptome analysis reveals autophagy as regulator of TGFβ/Smad-induced fibrogenesis in trabecular meshwork cells
Source: Sci Rep. 2019 Nov 6;9:16092. doi: 10.1038/s41598-019-52627-2 (PMC6834604; doi:10.1038/s41598-019-52627-2)
Supplement: Supplementary file 1 — Supplementary Dataset [file 41598_2019_52627_MOESM1_ESM.docx]

**SUPPLEMENTARY DATASET**

**Transcriptome analysis reveals autophagy as regulator of TGFβ/Smad-induced fibrogenesis in trabecular meshwork cells**

*April Nettesheim, Myoung Sup Shim, Josh Hirt, Paloma B. Liton (*)*

| **Table S1: List of known genes significantly up-regulated more than 1.5-fold in siAtg5/7- compared to siNC-transfected HTM cells** | | | |
| --- | --- | --- | --- |
| *Gene ID* | *Description* | *Fold* | *P-value* |
| IFI44L | interferon induced protein 44 like(IFI44L) | 4.97 | 3E-03 |
| IFIT1 | interferon induced protein with tetratricopeptide repeats 1(IFIT1) | 4.44 | 2E-04 |
| DDX60 | DExD/H-box helicase 60(DDX60) | 3.48 | 6E-04 |
| STX12 | syntaxin 12(STX12) | 3.09 | 3E-03 |
| PLCE1-AS1 | PLCE1 antisense RNA 1(PLCE1-AS1) | 2.89 | 4E-02 |
| USP41 | ubiquitin specific peptidase 41(USP41) | 2.88 | 1E-03 |
| MX1 | MX dynamin like GTPase 1(MX1) | 2.77 | 3E-03 |
| SLC33A1 | solute carrier family 33 member 1(SLC33A1) | 2.68 | 6E-03 |
| PAQR5 | progestin and adipoQ receptor family member 5(PAQR5) | 2.68 | 5E-02 |
| CDK14 | cyclin dependent kinase 14(CDK14) | 2.55 | 9E-04 |
| RSAD2 | radical S-adenosyl methionine domain containing 2(RSAD2) | 2.48 | 4E-04 |
| MESDC2 | mesoderm development candidate 2(MESDC2) | 2.47 | 1E-03 |
| SULF1 | sulfatase 1(SULF1) | 2.46 | 9E-04 |
| OAS2 | 2'-5'-oligoadenylate synthetase 2(OAS2) | 2.45 | 4E-02 |
| SAMHD1 | SAM and HD domain containing deoxynucleoside triphosphate triphosphohydrolase 1(SAMHD1) | 2.41 | 1E-03 |
| LMO3 | LIM domain only 3(LMO3) | 2.39 | 9E-03 |
| CEMIP | cell migration inducing hyaluronan binding protein(CEMIP) | 2.39 | 1E-02 |
| IFI44 | interferon induced protein 44(IFI44) | 2.37 | 2E-04 |
| ECM2 | extracellular matrix protein 2(ECM2) | 2.35 | 3E-02 |
| IFIT3 | interferon induced protein with tetratricopeptide repeats 3(IFIT3) | 2.34 | 8E-04 |
| FKBP14 | FK506 binding protein 14(FKBP14) | 2.33 | 1E-03 |
| SMOC1 | SPARC related modular calcium binding 1(SMOC1) | 2.30 | 5E-04 |
| DIO2 | deiodinase, iodothyronine type II(DIO2) | 2.27 | 1E-02 |
| ATM | ATM serine/threonine kinase(ATM) | 2.18 | 2E-02 |
| BAMBI | BMP and activin membrane bound inhibitor(BAMBI) | 2.17 | 8E-03 |
| FGL2 | fibrinogen like 2(FGL2) | 2.16 | 3E-03 |
| MAP2 | microtubule associated protein 2(MAP2) | 2.15 | 4E-02 |
| ISCA1P1 | iron-sulfur cluster assembly 1 pseudogene 1(ISCA1P1) | 2.14 | 2E-02 |
| OAS1 | 2'-5'-oligoadenylate synthetase 1(OAS1) | 2.13 | 2E-02 |
| TLR3 | toll like receptor 3(TLR3) | 2.12 | 5E-03 |
| MICAL2 | microtubule associated monooxygenase, calponin and LIM domain containing 2(MICAL2) | 2.10 | 5E-04 |
| TGFB2 | transforming growth factor beta 2(TGFB2) | 2.04 | 2E-02 |
| TSC22D1 | TSC22 domain family member 1(TSC22D1) | 2.04 | 4E-04 |
| MFN1 | mitofusin 1(MFN1) | 2.02 | 8E-03 |
| RNA5SP355 | RNA, 5S ribosomal pseudogene 355(RNA5SP355) | 2.02 | 3E-03 |
| PHACTR2 | phosphatase and actin regulator 2(PHACTR2) | 2.02 | 3E-04 |
| ENDOD1 | endonuclease domain containing 1(ENDOD1) | 2.01 | 2E-02 |
| ZKSCAN1 | zinc finger with KRAB and SCAN domains 1(ZKSCAN1) | 2.00 | 3E-03 |
| HSPA4L | heat shock protein family A (Hsp70) member 4 like(HSPA4L) | 2.00 | 4E-02 |
| LAP3P2 | leucine aminopeptidase 3 pseudogene 2(LAP3P2) | 2.00 | 1E-02 |
| AGGF1P4 | angiogenic factor with G-patch and FHA domains 1 pseudogene 4(AGGF1P4) | 2.00 | 5E-03 |
| ATF1 | activating transcription factor 1(ATF1) | 1.99 | 1E-02 |
| KLHL13 | kelch like family member 13(KLHL13) | 1.99 | 2E-02 |
| TMEM45A | transmembrane protein 45A(TMEM45A) | 1.99 | 2E-02 |
| HMCN1 | hemicentin 1(HMCN1) | 1.99 | 2E-02 |
| LAP3 | leucine aminopeptidase 3(LAP3) | 1.99 | 5E-03 |
| NIPSNAP3A | nipsnap homolog 3A(NIPSNAP3A) | 1.98 | 1E-02 |
| KCTD16 | potassium channel tetramerization domain containing 16(KCTD16) | 1.97 | 6E-03 |
| GANAB | glucosidase II alpha subunit(GANAB) | 1.96 | 5E-04 |
| TMEM2 | transmembrane protein 2(TMEM2) | 1.96 | 3E-03 |
| ANXA7 | annexin A7(ANXA7) | 1.96 | 1E-02 |
| RFPL4A | ret finger protein like 4A(RFPL4A) | 1.94 | 2E-02 |
| STX2 | syntaxin 2(STX2) | 1.94 | 4E-03 |
| RNASE4 | ribonuclease A family member 4(RNASE4) | 1.94 | 5E-03 |
| GBP1P1 | guanylate binding protein 1 pseudogene 1(GBP1P1) | 1.94 | 2E-03 |
| PDE8A | phosphodiesterase 8A(PDE8A) | 1.94 | 2E-03 |
| PDGFD | platelet derived growth factor D(PDGFD) | 1.93 | 1E-02 |
| ZNF426 | zinc finger protein 426(ZNF426) | 1.93 | 9E-03 |
| GLYATL3 | glycine-N-acyltransferase like 3(GLYATL3) | 1.92 | 1E-02 |
| PHF5A | PHD finger protein 5A(PHF5A) | 1.92 | 5E-03 |
| ITPR2 | inositol 1,4,5-trisphosphate receptor type 2(ITPR2) | 1.90 | 2E-03 |
| GBP1 | Guanylate Binding Protein 1 | 1.90 | 2E-03 |
| CYR61 | cysteine rich angiogenic inducer 61(CYR61) | 1.90 | 1E-02 |
| BTN3A3 | butyrophilin subfamily 3 member A3(BTN3A3) | 1.90 | 1E-03 |
| TPM4P1 | tropomyosin 4 pseudogene 1(TPM4P1) | 1.90 | 6E-04 |
| LDB2 | LIM domain binding 2(LDB2) | 1.89 | 2E-03 |
| C9orf78 | chromosome 9 open reading frame 78(C9orf78) | 1.89 | 5E-03 |
| TM2D1 | TM2 domain containing 1(TM2D1) | 1.89 | 2E-02 |
| FAT1 | FAT atypical cadherin 1(FAT1) | 1.89 | 6E-03 |
| SCG2 | secretogranin II(SCG2) | 1.88 | 1E-02 |
| NDUFA5P11 | NADH:ubiquinone oxidoreductase subunit A5 pseudogene 11(NDUFA5P11) | 1.88 | 4E-02 |
| RAB3GAP1 | RAB3 GTPase activating protein catalytic subunit 1(RAB3GAP1) | 1.88 | 2E-02 |
| GPC4 | glypican 4(GPC4) | 1.87 | 3E-02 |
| HHIP | hedgehog interacting protein(HHIP) | 1.87 | 4E-03 |
| PTN | pleiotrophin(PTN) | 1.86 | 1E-02 |
| STAT1 | signal transducer and activator of transcription 1(STAT1) | 1.86 | 5E-03 |
| GPR39 | G protein-coupled receptor 39(GPR39) | 1.86 | 3E-02 |
| CD164 | CD164 molecule(CD164) | 1.86 | 1E-02 |
| CYP1B1 | cytochrome P450 family 1 subfamily B member 1(CYP1B1) | 1.86 | 2E-03 |
| SNORD3D | small nucleolar RNA, C/D box 3D(SNORD3D) | 1.86 | 2E-02 |
| SAMD9 | sterile alpha motif domain containing 9(SAMD9) | 1.86 | 2E-03 |
| TOMM22P1 | TOMM22 pseudogene 1(TOMM22P1) | 1.86 | 2E-02 |
| BDH2P1 | 3-hydroxybutyrate dehydrogenase, type 2 pseudogene 1(BDH2P1) | 1.85 | 6E-03 |
| IKBKAP | inhibitor of kappa light polypeptide gene enhancer in B-cells, kinase complex-associated protein(IKBKAP) | 1.85 | 1E-02 |
| FOPNL | FGFR1OP N-terminal like(FOPNL) | 1.85 | 2E-02 |
| KDM2A | lysine demethylase 2A(KDM2A) | 1.85 | 2E-03 |
| IFIT2 | interferon induced protein with tetratricopeptide repeats 2(IFIT2) | 1.85 | 2E-02 |
| ERO1A | endoplasmic reticulum oxidoreductase 1 alpha(ERO1A) | 1.85 | 3E-03 |
| SHC4 | SHC adaptor protein 4(SHC4) | 1.85 | 1E-02 |
| FAM32BP | family with sequence similarity 32, member B(FAM32BP) | 1.84 | 1E-02 |
| NEGR1 | neuronal growth regulator 1(NEGR1) | 1.84 | 8E-04 |
| MGLL | monoglyceride lipase(MGLL) | 1.84 | 2E-03 |
| LYPD1 | LY6/PLAUR domain containing 1(LYPD1) | 1.83 | 5E-02 |
| CARD16 | caspase recruitment domain family member 16(CARD16) | 1.83 | 2E-02 |
| PLA2G12AP1 | phospholipase A2 group XIIA pseudogene 1(PLA2G12AP1) | 1.82 | 2E-02 |
| EIF4A2P1 | eukaryotic translation initiation factor 4A2 pseudogene 1(EIF4A2P1) | 1.82 | 1E-02 |
| BDH2 | 3-hydroxybutyrate dehydrogenase, type 2(BDH2) | 1.82 | 2E-03 |
| TFPI2 | tissue factor pathway inhibitor 2(TFPI2) | 1.81 | 6E-03 |
| ARHGAP42 | Rho GTPase activating protein 42(ARHGAP42) | 1.81 | 2E-02 |
| TMEM267 | transmembrane protein 267(TMEM267) | 1.81 | 3E-02 |
| MRFAP1 | Morf4 family associated protein 1(MRFAP1) | 1.81 | 3E-02 |
| ADAMTS5 | ADAM metallopeptidase with thrombospondin type 1 motif 5(ADAMTS5) | 1.80 | 5E-02 |
| OR6Q1 | olfactory receptor family 6 subfamily Q member 1 (gene/pseudogene)(OR6Q1) | 1.79 | 2E-03 |
| FAM173B | family with sequence similarity 173 member B(FAM173B) | 1.79 | 1E-02 |
| NUDCD3 | NudC domain containing 3(NUDCD3) | 1.79 | 1E-03 |
| NDUFA5 | NADH:ubiquinone oxidoreductase subunit A5(NDUFA5) | 1.79 | 3E-02 |
| ZNF721 | zinc finger protein 721(ZNF721) | 1.79 | 3E-03 |
| BTN3A1 | butyrophilin subfamily 3 member A1(BTN3A1) | 1.78 | 2E-03 |
| TRAPPC2 | trafficking protein particle complex 2(TRAPPC2) | 1.78 | 2E-02 |
| G3BP2 | G3BP stress granule assembly factor 2(G3BP2) | 1.78 | 2E-03 |
| PANDAR | promoter of CDKN1A antisense DNA damage activated RNA(PANDAR) | 1.78 | 1E-02 |
| FKBP7 | FK506 binding protein 7(FKBP7) | 1.77 | 3E-03 |
| TXNDC12 | thioredoxin domain containing 12(TXNDC12) | 1.77 | 2E-03 |
| RMDN1 | regulator of microtubule dynamics 1(RMDN1) | 1.77 | 3E-02 |
| EDNRA | endothelin receptor type A(EDNRA) | 1.76 | 6E-04 |
| GALNT5 | polypeptide N-acetylgalactosaminyltransferase 5(GALNT5) | 1.76 | 1E-02 |
| LIN7A | lin-7 homolog A, crumbs cell polarity complex component(LIN7A) | 1.76 | 5E-03 |
| LINC00628 | long intergenic non-protein coding RNA 628(LINC00628) | 1.76 | 3E-02 |
| PANCR | PITX2 adjacent non-coding RNA(PANCR) | 1.76 | 3E-03 |
| ID1 | inhibitor of DNA binding 1, HLH protein(ID1) | 1.76 | 9E-04 |
| MBTPS1 | membrane bound transcription factor peptidase, site 1(MBTPS1) | 1.76 | 1E-03 |
| TAF13 | TATA-box binding protein associated factor 13(TAF13) | 1.76 | 3E-02 |
| TPM4 | tropomyosin 4(TPM4) | 1.75 | 2E-03 |
| COL5A2 | collagen type V alpha 2 chain(COL5A2) | 1.75 | 2E-03 |
| EXTL2 | exostosin like glycosyltransferase 2(EXTL2) | 1.75 | 3E-03 |
| HAPLN1 | hyaluronan and proteoglycan link protein 1(HAPLN1) | 1.75 | 7E-03 |
| FAM3C2 | FAM3C pseudogene(FAM3C2) | 1.74 | 3E-02 |
| PDGFRL | platelet derived growth factor receptor like(PDGFRL) | 1.74 | 9E-03 |
| RWDD4 | RWD domain containing 4(RWDD4) | 1.74 | 4E-03 |
| NCK1 | NCK adaptor protein 1(NCK1) | 1.74 | 2E-02 |
| PRPS1 | phosphoribosyl pyrophosphate synthetase 1(PRPS1) | 1.73 | 2E-02 |
| ARHGAP42P1 | Rho GTPase activating protein 42 pseudogene 1(ARHGAP42P1) | 1.73 | 4E-02 |
| TMEM167B | transmembrane protein 167B(TMEM167B) | 1.73 | 8E-03 |
| DPY19L1 | dpy-19 like 1(DPY19L1) | 1.73 | 3E-02 |
| PDSS2 | prenyl (decaprenyl) diphosphate synthase, subunit 2(PDSS2) | 1.73 | 9E-03 |
| SLC46A3 | solute carrier family 46 member 3(SLC46A3) | 1.73 | 4E-02 |
| MARK3P3 | microtubule affinity regulating kinase 3 pseudogene 3(MARK3P3) | 1.73 | 2E-02 |
| MOSPD2 | motile sperm domain containing 2(MOSPD2) | 1.73 | 5E-03 |
| TRAPPC2B | trafficking protein particle complex 2B(TRAPPC2B) | 1.73 | 3E-03 |
| SNORD87 | small nucleolar RNA, C/D box 87(SNORD87) | 1.73 | 1E-02 |
| FAM210CP | family with sequence similarity 210, member B pseudogene(FAM210CP) | 1.72 | 7E-03 |
| MTATP6P11 | mitochondrially encoded ATP synthase 6 pseudogene 11(MTATP6P11) | 1.72 | 2E-02 |
| HSDL2 | hydroxysteroid dehydrogenase like 2(HSDL2) | 1.72 | 5E-03 |
| P4HA3 | prolyl 4-hydroxylase subunit alpha 3(P4HA3) | 1.72 | 5E-03 |
| IGFBP4 | insulin like growth factor binding protein 4(IGFBP4) | 1.72 | 9E-03 |
| NIPSNAP3B | nipsnap homolog 3B(NIPSNAP3B) | 1.72 | 2E-02 |
| GBP3 | guanylate binding protein 3(GBP3) | 1.72 | 5E-03 |
| UBE2L6 | ubiquitin conjugating enzyme E2 L6(UBE2L6) | 1.71 | 1E-02 |
| ENPP1 | ectonucleotide pyrophosphatase/phosphodiesterase 1(ENPP1) | 1.71 | 4E-03 |
| EIF4A2 | eukaryotic translation initiation factor 4A2(EIF4A2) | 1.71 | 2E-02 |
| S1PR1 | sphingosine-1-phosphate receptor 1(S1PR1) | 1.71 | 2E-02 |
| BTAF1 | B-TFIID TATA-box binding protein associated factor 1(BTAF1) | 1.70 | 2E-02 |
| HNRNPRP2 | heterogeneous nuclear ribonucleoprotein R pseudogene 2(HNRNPRP2) | 1.70 | 7E-03 |
| CALCOCO2 | calcium binding and coiled-coil domain 2(CALCOCO2) | 1.70 | 5E-03 |
| POLR3KP1 | RNA polymerase III subunit K pseudogene 1(POLR3KP1) | 1.70 | 2E-02 |
| LSAMP | limbic system-associated membrane protein(LSAMP) | 1.70 | 7E-03 |
| TRIM51FP | tripartite motif-containing 51F, pseudogene(TRIM51FP) | 1.70 | 3E-03 |
| PRUNE2 | prune homolog 2(PRUNE2) | 1.70 | 2E-02 |
| JPX_2 | JPX transcript, XIST activator conserved region 2 | 1.70 | 2E-03 |
| TMX3 | thioredoxin related transmembrane protein 3(TMX3) | 1.70 | 4E-02 |
| FGF5 | fibroblast growth factor 5(FGF5) | 1.70 | 3E-03 |
| DYNLT3P2 | dynein light chain Tctex-type 3 pseudogene 2(DYNLT3P2) | 1.70 | 3E-02 |
| MTM1 | myotubularin 1(MTM1) | 1.70 | 7E-03 |
| SLC5A3 | solute carrier family 5 member 3(SLC5A3) | 1.70 | 4E-02 |
| ZNF595 | zinc finger protein 595(ZNF595) | 1.69 | 2E-02 |
| ZNF675 | zinc finger protein 675(ZNF675) | 1.69 | 8E-03 |
| FTH1P25 | ferritin heavy chain 1 pseudogene 25(FTH1P25) | 1.69 | 2E-03 |
| DYX1C1-CCPG1 | DYX1C1-CCPG1 readthrough (NMD candidate)(DYX1C1-CCPG1) | 1.69 | 3E-02 |
| TMEM33 | transmembrane protein 33(TMEM33) | 1.69 | 1E-02 |
| TBC1D3F | TBC1 domain family member 3F(TBC1D3F) | 1.69 | 1E-02 |
| ALLC | allantoicase(ALLC) | 1.69 | 2E-03 |
| DBT | dihydrolipoamide branched chain transacylase E2(DBT) | 1.69 | 2E-02 |
| HMG20A | high mobility group 20A(HMG20A) | 1.69 | 1E-02 |
| MFSD1P1 | major facilitator superfamily domain containing 1 pseudogene 1(MFSD1P1) | 1.68 | 8E-03 |
| TRIM22 | tripartite motif containing 22(TRIM22) | 1.68 | 2E-03 |
| TLK2 | tousled like kinase 2(TLK2) | 1.68 | 3E-03 |
| BLOC1S6 | biogenesis of lysosomal organelles complex 1 subunit 6(BLOC1S6) | 1.68 | 1E-02 |
| CASP7 | caspase 7(CASP7) | 1.68 | 6E-03 |
| CDS2 | CDP-diacylglycerol synthase 2(CDS2) | 1.68 | 6E-03 |
| ZNF267 | zinc finger protein 267(ZNF267) | 1.67 | 5E-03 |
| PDCD6 | programmed cell death 6(PDCD6) | 1.67 | 1E-02 |
| SBDS | SBDS ribosome assembly guanine nucleotide exchange factor(SBDS) | 1.67 | 4E-02 |
| GBP2 | guanylate binding protein 2(GBP2) | 1.66 | 1E-02 |
| PCDH10 | protocadherin 10(PCDH10) | 1.66 | 1E-02 |
| APOL1 | apolipoprotein L1(APOL1) | 1.66 | 2E-02 |
| CCDC43 | coiled-coil domain containing 43(CCDC43) | 1.66 | 2E-02 |
| YIPF6 | Yip1 domain family member 6(YIPF6) | 1.66 | 2E-02 |
| ZNF664 | zinc finger protein 664(ZNF664) | 1.65 | 6E-03 |
| KDELC2 | KDEL motif containing 2(KDELC2) | 1.65 | 1E-02 |
| RPL22L1 | ribosomal protein L22 like 1(RPL22L1) | 1.65 | 5E-03 |
| DENND5A | DENN domain containing 5A(DENND5A) | 1.65 | 5E-03 |
| DYNLT3 | dynein light chain Tctex-type 3(DYNLT3) | 1.65 | 2E-02 |
| PDE3A | phosphodiesterase 3A(PDE3A) | 1.65 | 7E-03 |
| MAPK6PS4 | mitogen-activated protein kinase 6 pseudogene 4(MAPK6PS4) | 1.65 | 3E-02 |
| EDEM3 | ER degradation enhancing alpha-mannosidase like protein 3(EDEM3) | 1.65 | 2E-02 |
| ZNF227 | zinc finger protein 227(ZNF227) | 1.64 | 5E-03 |
| TRIM69 | tripartite motif containing 69(TRIM69) | 1.64 | 2E-03 |
| UFSP2 | UFM1 specific peptidase 2(UFSP2) | 1.64 | 3E-02 |
| EIF4A2P4 | eukaryotic translation initiation factor 4A2 pseudogene 4(EIF4A2P4) | 1.64 | 2E-02 |
| EIF4A1P11 | eukaryotic translation initiation factor 4A1 pseudogene 11(EIF4A1P11) | 1.64 | 5E-02 |
| ITGAV | integrin subunit alpha V(ITGAV) | 1.64 | 4E-02 |
| SNRK | SNF related kinase(SNRK) | 1.64 | 2E-02 |
| TM2D3 | TM2 domain containing 3(TM2D3) | 1.64 | 3E-02 |
| INPP5F | inositol polyphosphate-5-phosphatase F(INPP5F) | 1.64 | 3E-02 |
| SNORD91B | small nucleolar RNA, C/D box 91B(SNORD91B) | 1.63 | 1E-02 |
| C5orf15 | chromosome 5 open reading frame 15(C5orf15) | 1.63 | 1E-03 |
| TMEM246-AS1 | TMEM246 antisense RNA 1(TMEM246-AS1) | 1.63 | 4E-02 |
| SETD7 | SET domain containing lysine methyltransferase 7(SETD7) | 1.63 | 4E-03 |
| ZNF45 | zinc finger protein 45(ZNF45) | 1.63 | 1E-02 |
| ZNF268 | zinc finger protein 268(ZNF268) | 1.63 | 1E-02 |
| TRAPPC6B | trafficking protein particle complex 6B(TRAPPC6B) | 1.63 | 4E-03 |
| TMEM117 | transmembrane protein 117(TMEM117) | 1.63 | 1E-02 |
| EXOC2 | Exocyst Complex Component 2 | 1.63 | 1E-02 |
| HMGN2P39 | high mobility group nucleosomal binding domain 2 pseudogene 39(HMGN2P39) | 1.63 | 2E-02 |
| TMEM41B | transmembrane protein 41B(TMEM41B) | 1.63 | 2E-03 |
| CFHR1 | complement factor H related 1(CFHR1) | 1.63 | 4E-02 |
| SNORD88A | small nucleolar RNA, C/D box 88A(SNORD88A) | 1.62 | 1E-02 |
| CCPG1 | cell cycle progression 1(CCPG1) | 1.62 | 3E-02 |
| SCN2A | sodium voltage-gated channel alpha subunit 2(SCN2A) | 1.62 | 2E-02 |
| TFRC | transferrin receptor(TFRC) | 1.62 | 2E-02 |
| DTX3L | deltex E3 ubiquitin ligase 3L(DTX3L) | 1.62 | 9E-03 |
| KCNRG | potassium channel regulator(KCNRG) | 1.61 | 2E-02 |
| NPBWR2 | neuropeptides B/W receptor 2(NPBWR2) | 1.61 | 1E-02 |
| GMNC | geminin coiled-coil domain containing(GMNC) | 1.61 | 5E-03 |
| ARHGAP42P3 | Rho GTPase activating protein 42 pseudogene 3(ARHGAP42P3) | 1.61 | 4E-02 |
| PARP9 | poly(ADP-ribose) polymerase family member 9(PARP9) | 1.61 | 8E-03 |
| RMDN2 | regulator of microtubule dynamics 2(RMDN2) | 1.60 | 1E-02 |
| XAF1 | XIAP associated factor 1(XAF1) | 1.60 | 3E-02 |
| ANG | angiogenin(ANG) | 1.60 | 8E-03 |
| ZNF610 | zinc finger protein 610(ZNF610) | 1.60 | 2E-03 |
| PERP | PERP, TP53 apoptosis effector(PERP) | 1.60 | 1E-02 |
| ISCA1 | iron-sulfur cluster assembly 1(ISCA1) | 1.60 | 1E-02 |
| SLC17A5 | solute carrier family 17 member 5(SLC17A5) | 1.60 | 4E-02 |
| RNU6-996P | RNA, U6 small nuclear 996, pseudogene(RNU6-996P) | 1.60 | 1E-02 |
| RPL7L1P8 | ribosomal protein L7 like 1 pseudogene 8(RPL7L1P8) | 1.60 | 2E-02 |
| B4GALT1 | beta-1,4-galactosyltransferase 1(B4GALT1) | 1.60 | 9E-03 |
| MRFAP1L1 | Morf4 family associated protein 1 like 1(MRFAP1L1) | 1.60 | 4E-02 |
| ZNF121 | zinc finger protein 121(ZNF121) | 1.60 | 2E-02 |
| XPC | XPC complex subunit, DNA damage recognition and repair factor(XPC) | 1.60 | 8E-03 |
| HMGN1P19 | high mobility group nucleosome binding domain 1 pseudogene 19(HMGN1P19) | 1.60 | 7E-03 |
| TXNDC15 | thioredoxin domain containing 15(TXNDC15) | 1.60 | 4E-02 |
| TLK2P1 | tousled like kinase 2 pseudogene 1(TLK2P1) | 1.59 | 2E-02 |
| TMEM39A | transmembrane protein 39A(TMEM39A) | 1.59 | 3E-03 |
| GLCE | glucuronic acid epimerase(GLCE) | 1.59 | 1E-02 |
| RBM18 | RNA binding motif protein 18(RBM18) | 1.59 | 2E-02 |
| SUCLA2P1 | succinate-CoA ligase ADP-forming beta subunit pseudogene 1(SUCLA2P1) | 1.59 | 2E-02 |
| NOLC1 | nucleolar and coiled-body phosphoprotein 1(NOLC1) | 1.59 | 7E-03 |
| UBQLN3 | ubiquilin 3(UBQLN3) | 1.59 | 3E-02 |
| IGFBP3 | insulin like growth factor binding protein 3(IGFBP3) | 1.59 | 5E-03 |
| MIR764 | microRNA 764(MIR764) | 1.59 | 6E-03 |
| YIPF4 | Yip1 domain family member 4(YIPF4) | 1.59 | 5E-02 |
| RCC2P6 | regulator of chromosome condensation 2 pseudogene 6(RCC2P6) | 1.59 | 5E-03 |
| ZNF813 | zinc finger protein 813(ZNF813) | 1.59 | 1E-02 |
| ZDHHC18 | zinc finger DHHC-type containing 18(ZDHHC18) | 1.59 | 6E-03 |
| CA5AP1 | carbonic anhydrase 5A pseudogene 1(CA5AP1) | 1.59 | 6E-03 |
| HDAC1 | histone deacetylase 1(HDAC1) | 1.59 | 7E-03 |
| IL7 | interleukin 7(IL7) | 1.59 | 1E-02 |
| PDE4D | phosphodiesterase 4D(PDE4D) | 1.58 | 1E-02 |
| DYNLT3P1 | dynein light chain Tctex-type 3 pseudogene 1(DYNLT3P1) | 1.58 | 3E-02 |
| COL3A1 | collagen type III alpha 1 chain(COL3A1) | 1.58 | 2E-03 |
| TMEM47 | transmembrane protein 47(TMEM47) | 1.58 | 6E-03 |
| EIF4A1P7 | eukaryotic translation initiation factor 4A1 pseudogene 7(EIF4A1P7) | 1.58 | 4E-02 |
| HAS2 | hyaluronan synthase 2(HAS2) | 1.58 | 2E-02 |
| OLFML2B | olfactomedin like 2B(OLFML2B) | 1.58 | 4E-03 |
| CMKLR1 | chemerin chemokine-like receptor 1(CMKLR1) | 1.58 | 4E-02 |
| SBDSP1 | Shwachman-Bodian-Diamond syndrome pseudogene 1(SBDSP1) | 1.58 | 4E-02 |
| DPP4 | dipeptidyl peptidase 4(DPP4) | 1.58 | 2E-02 |
| LMBRD1 | LMBR1 domain containing 1(LMBRD1) | 1.58 | 2E-02 |
| WDR63 | WD repeat domain 63(WDR63) | 1.57 | 4E-03 |
| PCOLCE2 | procollagen C-endopeptidase enhancer 2(PCOLCE2) | 1.57 | 6E-03 |
| SNORD95 | small nucleolar RNA, C/D box 95(SNORD95) | 1.57 | 2E-02 |
| RPSAP16 | ribosomal protein SA pseudogene 16(RPSAP16) | 1.57 | 1E-02 |
| EIF4A2P2 | eukaryotic translation initiation factor 4A2 pseudogene 2(EIF4A2P2) | 1.57 | 4E-02 |
| RNA5SP322 | RNA, 5S ribosomal pseudogene 322(RNA5SP322) | 1.57 | 1E-02 |
| CFHR3 | complement factor H related 3(CFHR3) | 1.57 | 3E-02 |
| POLR2C | RNA polymerase II subunit C(POLR2C) | 1.56 | 7E-03 |
| AHCYL2 | adenosylhomocysteinase like 2(AHCYL2) | 1.56 | 4E-03 |
| OR8A3P | olfactory receptor family 8 subfamily A member 3 pseudogene(OR8A3P) | 1.56 | 1E-02 |
| FAM27C | family with sequence similarity 27 member C(FAM27C) | 1.56 | 6E-03 |
| SPTLC1 | serine palmitoyltransferase long chain base subunit 1(SPTLC1) | 1.56 | 3E-02 |
| TMED9 | transmembrane p24 trafficking protein 9(TMED9) | 1.56 | 4E-03 |
| CDH2 | cadherin 2(CDH2) | 1.55 | 5E-03 |
| SNORA8 | small nucleolar RNA, H/ACA box 8(SNORA8) | 1.55 | 2E-02 |
| TOMM22 | translocase of outer mitochondrial membrane 22(TOMM22) | 1.55 | 2E-02 |
| SOCS5P3 | suppressor of cytokine signaling 5 pseudogene 3(SOCS5P3) | 1.55 | 8E-03 |
| OPA1-AS1 | OPA1 antisense RNA 1(OPA1-AS1) | 1.55 | 4E-02 |
| NDUFB5 | NADH:ubiquinone oxidoreductase subunit B5(NDUFB5) | 1.55 | 2E-02 |
| SLC39A9 | solute carrier family 39 member 9(SLC39A9) | 1.55 | 4E-03 |
| ZNF91 | zinc finger protein 91(ZNF91) | 1.55 | 3E-02 |
| ANXA4 | annexin A4(ANXA4) | 1.55 | 1E-02 |
| ZNF808 | zinc finger protein 808(ZNF808) | 1.55 | 3E-02 |
| SLC39A7 | solute carrier family 39 member 7(SLC39A7) | 1.54 | 2E-02 |
| CYCS | cytochrome c, somatic(CYCS) | 1.54 | 4E-02 |
| EIF4A1P4 | eukaryotic translation initiation factor 4A1 pseudogene 4(EIF4A1P4) | 1.54 | 4E-02 |
| STAT2 | signal transducer and activator of transcription 2(STAT2) | 1.54 | 1E-02 |
| MFSD1 | major facilitator superfamily domain containing 1(MFSD1) | 1.54 | 1E-02 |
| ZNF480 | zinc finger protein 480(ZNF480) | 1.54 | 5E-03 |
| GUCY1A3 | guanylate cyclase 1 soluble subunit alpha(GUCY1A3) | 1.54 | 3E-03 |
| HTT-AS1_1 | HTT antisense RNA 1 conserved region 1 | 1.54 | 3E-02 |
| GPR176 | G protein-coupled receptor 176(GPR176) | 1.54 | 2E-02 |
| DSE | dermatan sulfate epimerase(DSE) | 1.54 | 9E-03 |
| ERG | ERG, ETS transcription factor(ERG) | 1.54 | 6E-03 |
| COX15 | COX15, cytochrome c oxidase assembly homolog(COX15) | 1.54 | 3E-02 |
| PLPP1 | phospholipid phosphatase 1(PLPP1) | 1.54 | 6E-03 |
| PPIC | peptidylprolyl isomerase C(PPIC) | 1.53 | 2E-02 |
| FAM32A | family with sequence similarity 32 member A(FAM32A) | 1.53 | 2E-02 |
| RNASEL | ribonuclease L(RNASEL) | 1.53 | 2E-02 |
| ZDHHC7 | zinc finger DHHC-type containing 7(ZDHHC7) | 1.53 | 1E-02 |
| NDUFS2 | NADH:ubiquinone oxidoreductase core subunit S2(NDUFS2) | 1.53 | 7E-03 |
| DPY19L3 | dpy-19 like 3 (C. elegans)(DPY19L3) | 1.53 | 1E-02 |
| DNAJB9 | DnaJ heat shock protein family (Hsp40) member B9(DNAJB9) | 1.53 | 4E-02 |
| KIRREL3-AS1 | KIRREL3 antisense RNA 1(KIRREL3-AS1) | 1.53 | 2E-02 |
| MIR1180 | microRNA 1180(MIR1180) | 1.53 | 4E-02 |
| RCC2P3 | regulator of chromosome condensation 2 pseudogene 3(RCC2P3) | 1.53 | 1E-02 |
| LONP2 | lon peptidase 2, peroxisomal(LONP2) | 1.53 | 2E-02 |
| RPS2P39 | ribosomal protein S2 pseudogene 39(RPS2P39) | 1.53 | 2E-02 |
| MAPK6PS3 | mitogen-activated protein kinase 6 pseudogene 3(MAPK6PS3) | 1.53 | 3E-02 |
| IFIT5 | interferon induced protein with tetratricopeptide repeats 5(IFIT5) | 1.52 | 4E-02 |
| ARL14EP | ADP ribosylation factor like GTPase 14 effector protein(ARL14EP) | 1.52 | 5E-02 |
| MAN1B1-AS1 | MAN1B1 antisense RNA 1 (head to head)(MAN1B1-AS1) | 1.52 | 4E-02 |
| MGAT5 | mannosyl (alpha-1,6-)-glycoprotein beta-1,6-N-acetyl-glucosaminyltransferase(MGAT5) | 1.52 | 2E-03 |
| ZNF33A | zinc finger protein 33A(ZNF33A) | 1.52 | 3E-02 |
| IGFBP5 | insulin like growth factor binding protein 5(IGFBP5) | 1.52 | 4E-02 |
| ZNF701 | zinc finger protein 701(ZNF701) | 1.52 | 2E-02 |
| ADAMTS9 | ADAM metallopeptidase with thrombospondin type 1 motif 9(ADAMTS9) | 1.52 | 3E-02 |
| DDX28 | DEAD-box helicase 28(DDX28) | 1.52 | 1E-02 |
| KLHL15 | kelch like family member 15(KLHL15) | 1.52 | 5E-02 |
| ZNF254 | zinc finger protein 254(ZNF254) | 1.52 | 2E-02 |
| FPGT | fucose-1-phosphate guanylyltransferase(FPGT) | 1.52 | 3E-02 |
| CHRNA6 | cholinergic receptor nicotinic alpha 6 subunit(CHRNA6) | 1.52 | 2E-02 |
| ARHGEF9 | Cdc42 guanine nucleotide exchange factor 9(ARHGEF9) | 1.52 | 4E-03 |
| FUT11 | fucosyltransferase 11(FUT11) | 1.52 | 4E-03 |
| MEIS1-AS3 | MEIS1 antisense RNA 3(MEIS1-AS3) | 1.52 | 3E-02 |
| CYP1B1-AS1 | CYP1B1 antisense RNA 1(CYP1B1-AS1) | 1.52 | 5E-03 |
| TMEM216 | transmembrane protein 216(TMEM216) | 1.52 | 1E-02 |
| SEC13P1 | SEC13 homolog, nuclear pore and COPII coat complex component pseudogene 1(SEC13P1) | 1.52 | 2E-02 |
| SNORD35A | small nucleolar RNA, C/D box 35A(SNORD35A) | 1.51 | 1E-02 |
| SDF2 | stromal cell derived factor 2(SDF2) | 1.51 | 1E-02 |
| MAPK6 | mitogen-activated protein kinase 6(MAPK6) | 1.51 | 1E-02 |
| DOCK1 | dedicator of cytokinesis 1(DOCK1) | 1.51 | 2E-02 |
| FGF7 | fibroblast growth factor 7(FGF7) | 1.51 | 6E-03 |
| MIR885 | microRNA 885(MIR885) | 1.51 | 2E-02 |
| RCC2P7 | regulator of chromosome condensation 2 pseudogene 7(RCC2P7) | 1.51 | 5E-03 |
| MR1 | major histocompatibility complex, class I-related(MR1) | 1.51 | 2E-02 |
| RYKP1 | receptor-like tyrosine kinase pseudogene 1(RYKP1) | 1.51 | 3E-02 |
| RARRES2P10 | retinoic acid receptor responder 2 pseudogene 10(RARRES2P10) | 1.51 | 7E-03 |
| SLC12A2 | solute carrier family 12 member 2(SLC12A2) | 1.51 | 5E-02 |
| CFH | complement factor H(CFH) | 1.51 | 2E-02 |
| SLC39A8 | solute carrier family 39 member 8(SLC39A8) | 1.51 | 1E-02 |
| LINC01520 | long intergenic non-protein coding RNA 1520(LINC01520) | 1.51 | 1E-02 |
| DZIP3 | DAZ interacting zinc finger protein 3(DZIP3) | 1.50 | 4E-02 |
| RN7SL110P | RNA, 7SL, cytoplasmic 110, pseudogene(RN7SL110P) | 1.50 | 1E-02 |
| CRYAB | crystallin alpha B(CRYAB) | 1.50 | 1E-02 |
| TIPRL | TOR signaling pathway regulator(TIPRL) | 1.50 | 5E-02 |
| PRDX4 | peroxiredoxin 4(PRDX4) | 1.50 | 3E-03 |
| ZNF253 | zinc finger protein 253(ZNF253) | 1.50 | 2E-02 |
| OGFR | opioid growth factor receptor(OGFR) | 1.50 | 6E-03 |
| LINC00371 | long intergenic non-protein coding RNA 371(LINC00371) | 1.50 | 1E-02 |
| TCEB3 | transcription elongation factor B subunit 3(TCEB3) | 1.50 | 1E-02 |
| SPCS3 | signal peptidase complex subunit 3(SPCS3) | 1.50 | 4E-02 |
| PTGS1 | prostaglandin-endoperoxide synthase 1(PTGS1) | 1.50 | 2E-02 |
| CALCRL | calcitonin receptor like receptor(CALCRL) | 1.50 | 5E-02 |
| PKD2 | polycystin 2, transient receptor potential cation channel(PKD2) | 1.50 | 9E-03 |

| **Table S2: List of known genes significantly down-regulated more than 1.5-fold in non-stretched siAtg5/7- compared to siNC-transfected HTM cells** | | | |
| --- | --- | --- | --- |
| *Gene ID* | *Description* | *Fold* | *P-value* |
| RN7SL303P | RNA, 7SL, cytoplasmic 303, pseudogene(RN7SL303P) | -17.40 | 5E-02 |
| EIF2B5-AS1 | EIF2B5 antisense RNA 1(EIF2B5-AS1) | -11.34 | 4E-02 |
| TMEM238 | transmembrane protein 238(TMEM238) | -3.31 | 4E-02 |
| PLAC9 | placenta specific 9(PLAC9) | -3.24 | 4E-02 |
| MTND4P23 | mitochondrially encoded NADH:ubiquinone oxidoreductase core subunit 4 pseudogene 23(MTND4P23) | -3.01 | 2E-02 |
| ATG5 | Autophagy Related 5 | -2.69 | 4.6E-05 |
| MTND5P2 | mitochondrially encoded NADH:ubiquinone oxidoreductase core subunit 5 pseudogene 2(MTND5P2) | -2.64 | 4E-03 |
| KRTAP2-3 | keratin associated protein 2-3(KRTAP2-3) | -2.59 | 4E-03 |
| MTND4P21 | mitochondrially encoded NADH:ubiquinone oxidoreductase core subunit 4 pseudogene 21(MTND4P21) | -2.54 | 2E-03 |
| AGR2 | anterior gradient 2, protein disulphide isomerase family member(AGR2) | -2.42 | 2E-03 |
| MTCO1P28 | mitochondrially encoded cytochrome c oxidase I pseudogene 28(MTCO1P28) | -2.38 | 1E-02 |
| SCAANT1 | SCA7/ATXN7 antisense RNA 1(SCAANT1) | -2.38 | 1E-02 |
| MTND5P4 | mitochondrially encoded NADH:ubiquinone oxidoreductase core subunit 5 pseudogene 4(MTND5P4) | -2.36 | 2E-02 |
| CFL1P7 | cofilin 1 pseudogene 7(CFL1P7) | -2.34 | 2E-03 |
| PROSER3 | proline and serine rich 3(PROSER3) | -2.31 | 3E-02 |
| MTATP6P25 | mitochondrially encoded ATP synthase 6 pseudogene 25(MTATP6P25) | -2.19 | 4E-02 |
| SNRPCP18 | small nuclear ribonucleoprotein polypeptide C pseudogene 18(SNRPCP18) | -2.17 | 1E-02 |
| HOXB7 | homeobox B7(HOXB7) | -2.17 | 3E-02 |
| HMGA2 | high mobility group AT-hook 2(HMGA2) | -2.16 | 3E-04 |
| SPX | Spexin Hormone | -2.16 | 1.5E-02 |
| FRMPD1 | FERM and PDZ domain containing 1(FRMPD1) | -2.16 | 3E-02 |
| MTCYBP13 | mitochondrially encoded cytochrome b pseudogene 13(MTCYBP13) | -2.16 | 4E-02 |
| RN7SKP187 | RNA, 7SK Small Nuclear Pseudogene 187 | -2.09 | 2.9E-02 |
| RPS24P7 | ribosomal protein S24 pseudogene 7(RPS24P7) | -2.07 | 1E-02 |
| DLX5 | distal-less homeobox 5(DLX5) | -2.07 | 4E-02 |
| HBA2 | hemoglobin subunit alpha 2(HBA2) | -2.05 | 3E-02 |
| ACOT4 | acyl-CoA thioesterase 4(ACOT4) | -2.05 | 4E-03 |
| MIR4731 | microRNA 4731(MIR4731) | -2.02 | 5E-03 |
| MTRNR2L3 | MT-RNR2-like 3(MTRNR2L3) | -2.01 | 2E-02 |
| MTND4P9 | mitochondrially encoded NADH:ubiquinone oxidoreductase core subunit 4 pseudogene 9(MTND4P9) | -2.01 | 3E-02 |
| SUMO2P13 | SUMO2 pseudogene 13(SUMO2P13) | -2.00 | 4E-02 |
| FTH1P24 | ferritin heavy chain 1 pseudogene 24(FTH1P24) | -1.99 | 5E-03 |
| MTCYBP22 | mitochondrially encoded cytochrome b pseudogene 22(MTCYBP22) | -1.99 | 2E-03 |
| MTND2P24 | mitochondrially encoded NADH:ubiquinone oxidoreductase core subunit 2 pseudogene 24(MTND2P24) | -1.99 | 1E-02 |
| MTND3P10 | mitochondrially encoded NADH:ubiquinone oxidoreductase core subunit 3 pseudogene 10(MTND3P10) | -1.99 | 2E-02 |
| MTCO3P22 | mitochondrially encoded cytochrome c oxidase III pseudogene 22(MTCO3P22) | -1.98 | 4E-03 |
| MTCYBP20 | mitochondrially encoded cytochrome b pseudogene 20(MTCYBP20) | -1.98 | 3E-02 |
| MTCO1P2 | mitochondrially encoded cytochrome c oxidase I pseudogene 2(MTCO1P2) | -1.97 | 2E-02 |
| LINC00226 | long intergenic non-protein coding RNA 226(LINC00226) | -1.97 | 1E-02 |
| OR4A21P | olfactory receptor family 4 subfamily A member 21 pseudogene(OR4A21P) | -1.96 | 4E-03 |
| EVI2B | ecotropic viral integration site 2B(EVI2B) | -1.95 | 5E-02 |
| MTCYBP10 | mitochondrially encoded cytochrome b pseudogene 10(MTCYBP10) | -1.93 | 4E-02 |
| FAM27D1 | family with sequence similarity 27 member D1(FAM27D1) | -1.92 | 4E-02 |
| AREL1 | Apoptosis Resistant E3 Ubiquitin Protein Ligase 1 | -1.91 | 4.9E-04 |
| HOXA11 | homeobox A11(HOXA11) | -1.91 | 1E-02 |
| RD3 | retinal degeneration 3(RD3) | -1.91 | 4E-02 |
| HOXA11-AS1_6 | HOXA11 antisense RNA 1 conserved region 6 | -1.91 | 6E-03 |
| RNY3P14 | RNA, Ro-associated Y3 pseudogene 14(RNY3P14) | -1.90 | 6E-03 |
| PPIAP9 | peptidylprolyl isomerase A pseudogene 9(PPIAP9) | -1.90 | 4E-02 |
| EVX1 | even-skipped homeobox 1(EVX1) | -1.89 | 2E-02 |
| FAM212B-AS1 | FAM212B antisense RNA 1(FAM212B-AS1) | -1.89 | 2E-02 |
| CPA4 | Carboxypeptidase A4 | -1.89 | 3.0E-03 |
| BDKRB1 | B1 bradykinin receptor | -1.88 | 3.3E-04 |
| CENPO | centromere protein O(CENPO) | -1.87 | 4E-02 |
| ZNF256 | zinc finger protein 256(ZNF256) | -1.87 | 4E-02 |
| RPL23AP63 | ribosomal protein L23a pseudogene 63(RPL23AP63) | -1.85 | 1E-02 |
| IGLV1-44 | immunoglobulin lambda variable 1-44(IGLV1-44) | -1.85 | 2E-02 |
| VMO1 | vitelline membrane outer layer 1 homolog(VMO1) | -1.84 | 8E-04 |
| MTCO3P24 | mitochondrially encoded cytochrome c oxidase III pseudogene 24(MTCO3P24) | -1.83 | 8E-03 |
| ZFHX4 | zinc finger homeobox 4(ZFHX4) | -1.83 | 4E-02 |
| RPL17P11 | ribosomal protein L17 pseudogene 11(RPL17P11) | -1.83 | 4E-02 |
| CRYGGP | crystallin gamma G, pseudogene(CRYGGP) | -1.82 | 3E-02 |
| KRTAP2-4 | keratin associated protein 5-10(KRTAP5-10) | -1.82 | 3E-02 |
| IGHJ1 | immunoglobulin heavy joining 1(IGHJ1) | -1.82 | 1E-02 |
| IGHVII-49-1 | immunoglobulin heavy variable (II)-49-1 (pseudogene)(IGHVII-49-1) | -1.81 | 7E-03 |
| RPL15P1 | ribosomal protein L15 pseudogene 1(RPL15P1) | -1.81 | 3E-03 |
| ATG7 | Autophagy Related 7 | -1.81 | 3.5E-04 |
| MTRNR2L2 | MT-RNR2-like 2(MTRNR2L2) | -1.80 | 5E-03 |
| STK31 | serine/threonine kinase 31(STK31) | -1.80 | 3E-02 |
| RPS27P23 | ribosomal protein S27 pseudogene 23(RPS27P23) | -1.79 | 2E-02 |
| OOSP2 | oocyte secreted protein 2(OOSP2) | -1.79 | 9E-03 |
| GAPDHP28 | glyceraldehyde 3 phosphate dehydrogenase pseudogene 28(GAPDHP28) | -1.79 | 5E-02 |
| GAPDHP75 | glyceraldehyde-3-phosphate dehydrogenase pseudogene 75(GAPDHP75) | -1.79 | 8E-03 |
| KRTAP2-2 | keratin associated protein 2-2(KRTAP2-2) | -1.79 | 2E-02 |
| EIF4EBP2P3 | Eukaryotic Translation Initiation Factor 4E Binding Protein 2 Pseudogene 3 | -1.79 | 4.3E-03 |
| RNU4-44P | RNA, U4 small nuclear 44, pseudogene(RNU4-44P) | -1.78 | 2E-02 |
| ATP6V1C1 | V-type proton ATPase subunit C 1 | -1.78 | 1.1E-02 |
| RN7SKP210 | RNA, 7SK small nuclear pseudogene 210(RN7SKP210) | -1.78 | 2E-02 |
| RPL21P24 | ribosomal protein L21 pseudogene 24(RPL21P24) | -1.77 | 3E-03 |
| GPRC5A | G protein-coupled receptor class C group 5 member A(GPRC5A) | -1.77 | 7E-03 |
| KRTAP10-8 | keratin associated protein 10-8(KRTAP10-8) | -1.77 | 2E-02 |
| HES7 | hes family bHLH transcription factor 7(HES7) | -1.77 | 2E-02 |
| PPIAL4G | peptidylprolyl isomerase A like 4G(PPIAL4G) | -1.77 | 3E-02 |
| MIR4313 | microRNA 4313(MIR4313) | -1.76 | 2E-02 |
| RNY1P10 | RNA, Ro-associated Y1 pseudogene 10(RNY1P10) | -1.76 | 2E-02 |
| CELF5 | CUGBP, Elav-like family member 5(CELF5) | -1.76 | 4E-02 |
| PTBP3 | Polypyrimidine Tract Binding Protein 3 | -1.76 | 2.8E-03 |
| MTCO2P22 | mitochondrially encoded cytochrome c oxidase II pseudogene 22(MTCO2P22) | -1.75 | 3E-02 |
| TSPY2 | testis specific protein, Y-linked 2(TSPY2) | -1.75 | 9E-03 |
| MAGI2-AS1 | MAGI2 antisense RNA 1(MAGI2-AS1) | -1.74 | 3E-03 |
| CALB1 | calbindin 1(CALB1) | -1.74 | 2E-02 |
| RN7SKP218 | RNA, 7SK small nuclear pseudogene 218(RN7SKP218) | -1.74 | 5E-03 |
| KLF7P1 | Kruppel Like Factor 7 Pseudogene 1 | -1.74 | 6.7E-03 |
| MCF2L-AS1 | MCF2L antisense RNA 1(MCF2L-AS1) | -1.73 | 4E-02 |
| RPL23AP94 | ribosomal protein L23a pseudogene 94(RPL23AP94) | -1.73 | 4E-02 |
| KRTAP9-10P | keratin associated protein 9-7(KRTAP9-7) | -1.73 | 2E-02 |
| SNX18P3 | sorting nexin 18 pseudogene 3(SNX18P3) | -1.73 | 1E-02 |
| RN7SKP98 | RNA, 7SK small nuclear pseudogene 98(RN7SKP98) | -1.73 | 4E-02 |
| RNU6-732P | RNA, U6 small nuclear 732, pseudogene(RNU6-732P) | -1.72 | 5E-02 |
| IGHV1-2 | immunoglobulin heavy variable 1-2(IGHV1-2) | -1.72 | 3E-02 |
| KRTAP9-7 | keratin associated protein 2-4(KRTAP2-4) | -1.72 | 9E-03 |
| MTCO1P7 | mitochondrially encoded cytochrome c oxidase I pseudogene 7(MTCO1P7) | -1.71 | 2E-02 |
| HIST1H4L | Histone Cluster 1 H4 Family Member L | -1.71 | 2.1E-02 |
| DMRT1 | doublesex and mab-3 related transcription factor 1(DMRT1) | -1.71 | 8E-03 |
| ESCO2 | establishment of sister chromatid cohesion N-acetyltransferase 2(ESCO2) | -1.71 | 3E-03 |
| LINC00977 | long intergenic non-protein coding RNA 977(LINC00977) | -1.70 | 5E-03 |
| PRAMEF15 | PRAME family member 15(PRAMEF15) | -1.70 | 3E-02 |
| PHLDA2 | Pleckstrin Homology Like Domain Family A Member 2 | -1.70 | 1.1E-02 |
| SSTR4 | somatostatin receptor 4(SSTR4) | -1.70 | 1E-02 |
| HOXA11-AS | HOXA11 antisense RNA(HOXA11-AS) | -1.70 | 9E-03 |
| SOX21 | SRY-box 21(SOX21) | -1.69 | 3E-02 |
| RN7SL48P | RNA, 7SL, cytoplasmic 48, pseudogene(RN7SL48P) | -1.69 | 2E-03 |
| RN7SL612P | RNA, 7SL, cytoplasmic 612, pseudogene(RN7SL612P) | -1.69 | 4E-02 |
| PPIAP27 | peptidylprolyl isomerase A pseudogene 27(PPIAP27) | -1.69 | 2E-02 |
| PROSER1 | proline and serine rich 1(PROSER1) | -1.69 | 2E-02 |
| GLYCAM1 | glycosylation dependent cell adhesion molecule 1 (pseudogene)(GLYCAM1) | -1.68 | 2E-02 |
| RNY1P12 | RNA, Ro-Associated Y1 Pseudogene 12 | -1.68 | 3.4E-03 |
| ACTA2-AS1 | ACTA2 Antisense RNA 1 | -1.68 | 6.4E-03 |
| GAPDHP23 | glyceraldehyde 3 phosphate dehydrogenase pseudogene 23(GAPDHP23) | -1.68 | 1E-02 |
| EIF4EBP2P2 | Eukaryotic Translation Initiation Factor 4E Binding Protein 2 Pseudogene 2 | -1.68 | 3.8E-03 |
| RPL12P22 | ribosomal protein L12 pseudogene 22(RPL12P22) | -1.68 | 5E-02 |
| WDR76 | WD repeat domain 76(WDR76) | -1.68 | 5E-03 |
| RN7SKP11 | RNA, 7SK small nuclear pseudogene 11(RN7SKP11) | -1.67 | 2E-02 |
| RN7SKP186 | RNA, 7SK small nuclear pseudogene 186(RN7SKP186) | -1.67 | 1E-02 |
| MYADML | myeloid associated differentiation marker like (pseudogene)(MYADML) | -1.67 | 1E-02 |
| PFN1P11 | profilin 1 pseudogene 11(PFN1P11) | -1.67 | 1E-02 |
| RN7SL570P | RNA, 7SL, cytoplasmic 570, pseudogene(RN7SL570P) | -1.66 | 2E-02 |
| RPS27P5 | ribosomal protein S27 pseudogene 5(RPS27P5) | -1.66 | 6E-03 |
| MCM8 | minichromosome maintenance 8 homologous recombination repair factor(MCM8) | -1.66 | 4E-03 |
| NT5CP2 | 5',3'-nucleotidase, cytosolic pseudogene 2(NT5CP2) | -1.66 | 1E-02 |
| COA6 | Cytochrome c oxidase assembly factor 6 homolog | -1.66 | 4.7E-02 |
| NEDD4 | neural precursor cell expressed, developmentally down-regulated 4, E3 ubiquitin protein ligase(NEDD4) | -1.66 | 2E-02 |
| UBA52P3 | ubiquitin A-52 residue ribosomal protein fusion product 1 pseudogene 3(UBA52P3) | -1.66 | 4E-02 |
| SIRPD | signal regulatory protein delta(SIRPD) | -1.65 | 3E-03 |
| MTCO3P9 | mitochondrially encoded cytochrome c oxidase III pseudogene 9(MTCO3P9) | -1.65 | 4E-02 |
| MIR29A | microRNA 29a(MIR29A) | -1.65 | 6E-03 |
| FGF3 | fibroblast growth factor 3(FGF3) | -1.65 | 3E-03 |
| TXNP7 | thioredoxin pseudogene 7(TXNP7) | -1.65 | 1E-02 |
| RNY1P13 | RNA, Ro-Associated Y1 Pseudogene 13 | -1.64 | 1.3E-02 |
| CDK2AP2P2 | Cyclin Dependent Kinase 2 Associated Protein 2 Pseudogene 2 | -1.64 | 4.5E-02 |
| RPL10P15 | ribosomal protein L10 pseudogene 15(RPL10P15) | -1.64 | 5E-03 |
| LINC00264 | long intergenic non-protein coding RNA 264(LINC00264) | -1.64 | 2E-02 |
| GAPDHP47 | glyceraldehyde 3 phosphate dehydrogenase pseudogene 47(GAPDHP47) | -1.64 | 5E-03 |
| SLC35B4 | Solute Carrier Family 35 Member B4 | -1.64 | 7.9E-03 |
| IGHV2OR16-5 | immunoglobulin heavy variable 2/OR16-5 (non-functional)(IGHV2OR16-5) | -1.64 | 1E-02 |
| MACROD2-AS1 | MACROD2 antisense RNA 1(MACROD2-AS1) | -1.64 | 1E-02 |
| HMGB2 | High Mobility Group Box 2 | -1.63 | 3.0E-02 |
| FBXO36 | F-box protein 36(FBXO36) | -1.63 | 3E-02 |
| RPL35AP32 | ribosomal protein L35a pseudogene 32(RPL35AP32) | -1.63 | 5E-02 |
| MIR128-2 | microRNA 128-2(MIR128-2) | -1.63 | 2E-02 |
| NPM1P45 | nucleophosmin 1 pseudogene 45(NPM1P45) | -1.63 | 3E-02 |
| RPL23AP41 | ribosomal protein L23a pseudogene 41(RPL23AP41) | -1.63 | 2E-02 |
| LIF | leukemia inhibitory factor(LIF) | -1.63 | 1E-02 |
| RN7SL159P | RNA, 7SL, cytoplasmic 159, pseudogene(RN7SL159P) | -1.63 | 4E-03 |
| PAQR9 | progestin and adipoQ receptor family member 9(PAQR9) | -1.63 | 2E-02 |
| RNY1P5 | RNA, Ro-associated Y1 pseudogene 5(RNY1P5) | -1.63 | 3E-02 |
| ZNF77 | zinc finger protein 77(ZNF77) | -1.63 | 3E-02 |
| RPL29P23 | Ribosomal Protein L29 Pseudogene 23 | -1.62 | 1.6E-02 |
| KRT40 | keratin 40(KRT40) | -1.62 | 3E-02 |
| OR5M11 | olfactory receptor family 5 subfamily M member 11(OR5M11) | -1.62 | 2E-02 |
| SPDEF | SAM pointed domain containing ETS transcription factor(SPDEF) | -1.62 | 2E-02 |
| PABPC4L | poly(A) binding protein cytoplasmic 4 like(PABPC4L) | -1.62 | 2E-02 |
| TBCD | Tubulin Folding Cofactor D | -1.62 | 6.2E-04 |
| VEZT | Vezatin, Adherens Junctions Transmembrane Protein | -1.62 | 3.7E-03 |
| ANKRD35 | ankyrin repeat domain 35(ANKRD35) | -1.62 | 2E-02 |
| UBE2L5P | ubiquitin conjugating enzyme E2 L5, pseudogene(UBE2L5P) | -1.62 | 4E-03 |
| CXCR6 | C-X-C motif chemokine receptor 6(CXCR6) | -1.62 | 2E-02 |
| CACNA2D3-AS1 | CACNA2D3 antisense RNA 1(CACNA2D3-AS1) | -1.61 | 3E-02 |
| STEAP2-AS1 | STEAP2 antisense RNA 1(STEAP2-AS1) | -1.61 | 1E-02 |
| RPL23AP28 | ribosomal protein L23a pseudogene 28(RPL23AP28) | -1.61 | 3E-02 |
| PPIF | Peptidyl-prolyl cis-trans isomerase F, mitochondrial | -1.61 | 6.3E-04 |
| MTCYBP35 | MTCYBP35 mitochondrially encoded cytochrome b pseudogene 35 | -1.61 | 3E-02 |
| MTCYBP18 | mitochondrially encoded cytochrome b pseudogene 18(MTCYBP18) | -1.61 | 4E-02 |
| UBTFL7 | upstream binding transcription factor, RNA polymerase I-like 7 (pseudogene)(UBTFL7) | -1.61 | 7E-03 |
| SPRR2C | small proline rich protein 2C (pseudogene)(SPRR2C) | -1.61 | 3E-02 |
| KRTAP10-12 | keratin associated protein 10-12(KRTAP10-12) | -1.61 | 4E-02 |
| SUSD3 | sushi domain containing 3(SUSD3) | -1.61 | 9E-03 |
| NUTF2P4 | nuclear transport factor 2 pseudogene 4(NUTF2P4) | -1.61 | 3E-02 |
| SLC13A1 | solute carrier family 13 member 1(SLC13A1) | -1.61 | 9E-03 |
| EIF4EBP2 | Eukaryotic Translation Initiation Factor 4E Binding Protein 2 | -1.61 | 4.8E-03 |
| TMEM56 | transmembrane protein 56(TMEM56) | -1.61 | 2E-02 |
| POU4F1 | POU class 4 homeobox 1(POU4F1) | -1.60 | 1E-02 |
| GAPDHP64 | glyceraldehyde-3-phosphate dehydrogenase pseudogene 64(GAPDHP64) | -1.60 | 2E-02 |
| RPL10P5 | ribosomal protein L10 pseudogene 5(RPL10P5) | -1.60 | 5E-02 |
| MCM5 | minichromosome maintenance complex component 5(MCM5) | -1.60 | 3E-03 |
| WFDC2 | WAP four-disulfide core domain 2(WFDC2) | -1.60 | 3E-02 |
| RPL23AP31 | ribosomal protein L23a pseudogene 31(RPL23AP31) | -1.60 | 2E-02 |
| MIR4648 | microRNA 4648(MIR4648) | -1.60 | 3E-02 |
| CLEC4F | C-type lectin domain family 4 member F(CLEC4F) | -1.60 | 3E-02 |
| CEND1P1 | cell cycle exit and neuronal differentiation 1 pseudogene 1(CEND1P1) | -1.60 | 3E-02 |
| KCTD9P1 | potassium channel tetramerization domain containing 9 pseudogene 1(KCTD9P1) | -1.59 | 1E-02 |
| TMEM245 | Transmembrane Protein 245 | -1.59 | 5.1E-03 |
| UTF1 | undifferentiated embryonic cell transcription factor 1(UTF1) | -1.59 | 4E-02 |
| SLC7A5 | Solute Carrier Family 7 Member 5 | -1.59 | 2.2E-03 |
| ZNF877P | zinc finger protein 877, pseudogene(ZNF877P) | -1.59 | 2E-02 |
| RN7SL190P | RNA, 7SL, cytoplasmic 190, pseudogene(RN7SL190P) | -1.58 | 5E-03 |
| NPIPA5 | nuclear pore complex interacting protein family member A5(NPIPA5) | -1.58 | 4E-02 |
| CYP4F11 | cytochrome P450 family 4 subfamily F member 11(CYP4F11) | -1.58 | 4E-02 |
| HIST1H3A | Histone Cluster 1 H3 Family Member A | -1.58 | 4.1E-02 |
| STK32A | serine/threonine kinase 32A(STK32A) | -1.58 | 9E-03 |
| AGT | angiotensinogen(AGT) | -1.58 | 3E-02 |
| RPS23P1 | ribosomal protein S23 pseudogene 1(RPS23P1) | -1.58 | 2E-02 |
| KCNK15-AS1 | KCNK15 antisense RNA 1 (head to head)(KCNK15-AS1) | -1.58 | 1E-02 |
| SPATA4 | spermatogenesis associated 4(SPATA4) | -1.58 | 7E-03 |
| C2CD4C | C2 calcium dependent domain containing 4C(C2CD4C) | -1.58 | 5E-02 |
| RAB3A | RAB3A, member RAS oncogene family(RAB3A) | -1.58 | 1E-02 |
| MTRNR2L7 | MT-RNR2-like 7(MTRNR2L7) | -1.57 | 7E-03 |
| PFN1P9 | profilin 1 pseudogene 9(PFN1P9) | -1.57 | 9E-03 |
| GAPDHP33 | glyceraldehyde 3 phosphate dehydrogenase pseudogene 33(GAPDHP33) | -1.57 | 3E-02 |
| RBMXL2 | RNA binding motif protein, X-linked like 2(RBMXL2) | -1.57 | 2E-02 |
| POLR3G | RNA polymerase III subunit G(POLR3G) | -1.57 | 1E-02 |
| CDC42P4 | cell division cycle 42 pseudogene 4(CDC42P4) | -1.57 | 5E-03 |
| FAM219A | family with sequence similarity 219 member A(FAM219A) | -1.57 | 1E-02 |
| ZNF826P | zinc finger protein 826, pseudogene(ZNF826P) | -1.57 | 2E-02 |
| MTND1P3 | mitochondrially encoded NADH:ubiquinone oxidoreductase core subunit 1 pseudogene 3(MTND1P3) | -1.57 | 2E-02 |
| CYP2C8 | cytochrome P450 family 2 subfamily C member 8(CYP2C8) | -1.57 | 3E-02 |
| ATP2B4 | ATPase Plasma Membrane Ca2+ Transporting 4 | -1.57 | 8.5E-03 |
| HORMAD2-AS1 | HORMAD2 antisense RNA 1(HORMAD2-AS1) | -1.57 | 1E-02 |
| LINC00319 | long intergenic non-protein coding RNA 319(LINC00319) | -1.56 | 3E-02 |
| RGCC | regulator of cell cycle(RGCC) | -1.56 | 2E-02 |
| RIC3 | RIC3 acetylcholine receptor chaperone(RIC3) | -1.56 | 3E-02 |
| FGF10-AS1 | FGF10 antisense RNA 1(FGF10-AS1) | -1.56 | 1E-02 |
| ZNF774 | zinc finger protein 774(ZNF774) | -1.56 | 6E-03 |
| OSGIN1 | oxidative stress induced growth inhibitor 1(OSGIN1) | -1.56 | 8E-03 |
| ACTA2 | Actin, Alpha 2, Smooth Muscle, Aorta | -1.56 | 2.9E-03 |
| COLCA1 | colorectal cancer associated 1(COLCA1) | -1.56 | 3E-02 |
| RPL23AP27 | ribosomal protein L23a pseudogene 27(RPL23AP27) | -1.56 | 2E-02 |
| RPL23AP96 | ribosomal protein L23a pseudogene 96(RPL23AP96) | -1.55 | 3E-02 |
| FAM90A27P | family with sequence similarity 90 member A27, pseudogene(FAM90A27P) | -1.55 | 2E-02 |
| TUBAL3 | tubulin alpha like 3(TUBAL3) | -1.55 | 1E-02 |
| CCAT2 | colon cancer associated transcript 2 (non-protein coding)(CCAT2) | -1.55 | 3E-02 |
| TUBA3GP | tubulin alpha 3g pseudogene(TUBA3GP) | -1.55 | 5E-02 |
| RPL19P13 | ribosomal protein L19 pseudogene 13(RPL19P13) | -1.55 | 2E-02 |
| TGIF2P1 | TGFB induced factor homeobox 2 pseudogene 1(TGIF2P1) | -1.55 | 9E-03 |
| PPP3CC | Protein Phosphatase 3 Catalytic Subunit Gamma | -1.55 | 1.6E-03 |
| RNU6-16P | RNA, U6 small nuclear 16, pseudogene(RNU6-16P) | -1.55 | 2E-02 |
| GLOD5 | glyoxalase domain containing 5(GLOD5) | -1.55 | 3E-02 |
| GPR3 | G protein-coupled receptor 3(GPR3) | -1.55 | 4E-02 |
| SLC25A39P2 | SLC25A39 pseudogene 2(SLC25A39P2) | -1.55 | 2E-02 |
| LCE2A | late cornified envelope 2A(LCE2A) | -1.55 | 7E-03 |
| RNA5SP461 | RNA, 5S ribosomal pseudogene 461(RNA5SP461) | -1.54 | 3E-02 |
| MESTP1 | mesoderm specific transcript pseudogene 1(MESTP1) | -1.54 | 4E-02 |
| AC011290.5 | Thioredoxin Related Transmembrane Protein 1 Pseudogene | -1.54 | 2.6E-02 |
| RPL10P12 | ribosomal protein L10 pseudogene 12(RPL10P12) | -1.54 | 1E-02 |
| ACTG1P15 | actin gamma 1 pseudogene 15(ACTG1P15) | -1.54 | 1E-02 |
| RPL10P16 | ribosomal protein L10 pseudogene 16(RPL10P16) | -1.54 | 9E-03 |
| LINC00973 | long intergenic non-protein coding RNA 973(LINC00973) | -1.54 | 9E-03 |
| NCOR1P2 | nuclear receptor corepressor 1 pseudogene 2(NCOR1P2) | -1.54 | 3E-02 |
| RPL26P35 | ribosomal protein L26 pseudogene 35(RPL26P35) | -1.54 | 1E-02 |
| TMPRSS7 | transmembrane protease, serine 7(TMPRSS7) | -1.54 | 1E-02 |
| CCND2P1 | cyclin D2 pseudogene 1(CCND2P1) | -1.54 | 3E-03 |
| SENP1 | SUMO1/sentrin specific peptidase 1(SENP1) | -1.54 | 8E-03 |
| SNX2 | sorting nexin 2(SNX2) | -1.54 | 3E-03 |
| RPS27AP1 | ribosomal protein S27a pseudogene 1(RPS27AP1) | -1.54 | 2E-02 |
| FGF10 | fibroblast growth factor 10(FGF10) | -1.53 | 1E-02 |
| YBX2P1 | Y-box binding protein 2 pseudogene 1(YBX2P1) | -1.53 | 3E-02 |
| SPRR3 | small proline rich protein 3(SPRR3) | -1.53 | 1E-02 |
| BCDIN3D-AS1 | BCDIN3D antisense RNA 1(BCDIN3D-AS1) | -1.53 | 1E-02 |
| MIR1228 | microRNA 1228(MIR1228) | -1.53 | 5E-03 |
| FABP3P2 | fatty acid binding protein 3 pseudogene 2(FABP3P2) | -1.53 | 3E-02 |
| NKPD1 | NTPase, KAP family P-loop domain containing 1(NKPD1) | -1.53 | 5E-02 |
| SERPINE1 | Serpin Family E Member 1 | -1.52 | 1.0E-02 |
| GPR182 | G protein-coupled receptor 182(GPR182) | -1.52 | 3E-02 |
| LINC00868 | long intergenic non-protein coding RNA 868(LINC00868) | -1.52 | 4E-02 |
| MIR210 | microRNA 210 (MIR210) | -1.52 | 5E-02 |
| RPL10P11 | ribosomal protein L10 pseudogene 11(RPL10P11) | -1.52 | 4E-02 |
| CELSR3-AS1 | CELSR3 antisense RNA 1 (head to head)(CELSR3-AS1) | -1.52 | 1E-02 |
| RBPMS-AS1 | RBPMS antisense RNA 1(RBPMS-AS1) | -1.52 | 9E-03 |
| LPAR3 | lysophosphatidic acid receptor 3(LPAR3) | -1.52 | 4E-02 |
| GAPDHP26 | glyceraldehyde 3 phosphate dehydrogenase pseudogene 26(GAPDHP26) | -1.52 | 4E-03 |
| RNU4-20P | RNA, U4 Small Nuclear 20, Pseudogene | -1.52 | 3.1E-02 |
| TBXA2R | thromboxane A2 receptor(TBXA2R) | -1.52 | 3E-02 |
| SP7 | Sp7 transcription factor(SP7) | -1.52 | 2E-02 |
| TSPEAR-AS2 | TSPEAR antisense RNA 2(TSPEAR-AS2) | -1.52 | 9E-03 |
| CNN1 | Calponin 1 | -1.52 | 2.0E-02 |
| TONSL-AS1 | TONSL antisense RNA 1(TONSL-AS1) | -1.52 | 1E-02 |
| APOBEC3B | apolipoprotein B mRNA editing enzyme catalytic subunit 3B(APOBEC3B) | -1.52 | 6E-03 |
| CCND1 | cyclin D1(CCND1) | -1.52 | 3E-03 |
| DHRS2 | dehydrogenase/reductase 2(DHRS2) | -1.52 | 4E-03 |
| RPL7AP4 | ribosomal protein L7a pseudogene 4(RPL7AP4) | -1.51 | 2E-02 |
| ATP5J2P6 | ATP synthase, H+ transporting, mitochondrial Fo complex subunit F2 pseudogene 6(ATP5J2P6) | -1.51 | 1E-02 |
| MIR1972-2 | microRNA 1972-2(MIR1972-2) | -1.51 | 5E-02 |
| KRTAP5-10 | keratin associated protein 9-10, pseudogene(KRTAP9-10P) | -1.51 | 9E-03 |
| SPATC1L | spermatogenesis and centriole associated 1-like(SPATC1L) | -1.51 | 2E-02 |
| RFX8 | RFX family member 8, lacking RFX DNA binding domain(RFX8) | -1.51 | 2E-02 |
| MTND4P16 | mitochondrially encoded NADH:ubiquinone oxidoreductase core subunit 4 pseudogene 16(MTND4P16) | -1.51 | 2E-02 |
| IGLV3-6 | immunoglobulin lambda variable 3-6 (pseudogene)(IGLV3-6) | -1.51 | 1E-02 |
| RN7SL492P | RNA, 7SL, cytoplasmic 492, pseudogene(RN7SL492P) | -1.51 | 1E-02 |
| RPL36AP13 | ribosomal protein L36a pseudogene 13(RPL36AP13) | -1.51 | 4E-02 |
| ZNRF2P3 | zinc and ring finger 2 pseudogene 3(ZNRF2P3) | -1.51 | 2E-02 |
| GRK7 | G protein-coupled receptor kinase 7(GRK7) | -1.51 | 3E-02 |
| LRRC14B | leucine rich repeat containing 14B(LRRC14B) | -1.51 | 2E-02 |
| NKX3-2 | NK3 homeobox 2(NKX3-2) | -1.50 | 2E-02 |
| RPS4XP13 | ribosomal protein S4X pseudogene 13(RPS4XP13) | -1.50 | 7E-03 |
| MTRNR2L4 | MT-RNR2-like 4(MTRNR2L4) | -1.50 | 5E-02 |
| RPL23AP3 | ribosomal protein L23a pseudogene 3(RPL23AP3) | -1.50 | 3E-02 |
| RPS3AP41 | ribosomal protein S3a pseudogene 41(RPS3AP41) | -1.50 | 4E-02 |
