# Supplementary material for: Transcriptome analysis reveals autophagy as regulator of TGFβ/Smad-induced fibrogenesis in trabecular meshwork cells
Source: Sci Rep. 2019 Nov 6;9:16092. doi: 10.1038/s41598-019-52627-2 (PMC6834604; doi:10.1038/s41598-019-52627-2)

SUPPLEMENTAL MATERIAL

Transcriptome analysis reveals autophagy as regulator of TGFβ/Smad-induced fibrogenesis in trabecular meshwork cells

April Nettesheim, Myoung Sup Shim, Josh Hirt, Paloma B. Liton (*)

Supplemental Material to Figure 2: Levels of total TGFβ1 (latent and active) in culture media from siAtg5/7-transfected TM cells quantified by ELISA (Invitrogen) and normalized with total μg of corresponding whole cell lysates. n=3, t-test.

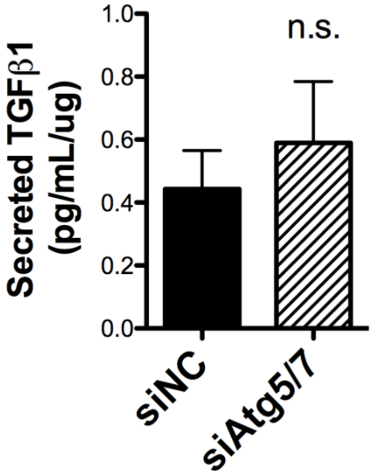

SUPLEMENTAL MATERIAL TO FIGURE 3

Exact mean values ±SD and statistical analysis

	SM-Fig. 3B: SMA Relative Protein Leves (% of siNC-Ctrl)															
	Ctrl			TGF vs Ctrl	siAtg5/7			siRNA vs TGF	siLC3			siRNA vs TGF	siSmad2/3			siRNA vs TGF
	Mean	*SD*	*n*	*pV (†)*	*Mean*	*SD*	*n*	*pV (‡)*	*Mean*	*SD*	*n*	*pV (‡)*	*Mean*	*SD*	*n*	*pV (‡)*
**Ctrl**	100.00	0.00	5		64.00	15.00	5		78.50	6.61	4		58.25	13.12	4	
**TGFβ1**	241.60	127.64	5	0.05	85.40	21.38	5	<0.01	104.25	16.17	4	<0.05	61.50	8.96	4	<0.01
**TGFβ2**	290.60	147.48	5	0.05	124.60	46.78	5	<0.01	114.25	26.32	4	<0.01	61.25	30.98	4	<0.001

(†) One-way ANOVA (p=0.05)
(‡) Two-Way Anova (siRNA: p<0.0001; TGF: p=0.005)

	SM-Fig. 3C: Intracellular FN1 Relative Protein Leves (% of siNC-Ctrl)															
	Ctrl			TGF vs Ctrl	siAtg5/7			siRNA vs TGF	siLC3			siRNA vs TGF	siSmad2/3			siRNA vs TGF
	Mean	*SD*	*n*	*pV (†)*	*Mean*	*SD*	*n*	*pV (‡)*	*Mean*	*SD*	*n*	*pV (‡)*	*Mean*	*SD*	*n*	*pV (‡)*
**Ctrl**	100.00	0.00	5		50.25	41.92	4		75.00	33.68	5		20.80	21.30	5	<0.05
**TGFβ1**	135.00	73.43	5	n.s.	46.00	21.21	4	<0.01	69.80	13.52	5	n.s.	20.40	16.16	5	p<0.001
**TGFβ2**	111.80	38.44	5	n.s.	37.00	35.01	4	<0.05	69.80	50.43	5	n.s.	27.40	24.28	5	p<0.01

(†) One-way ANOVA (p=n.s)
(‡) Two-Way Anova (siRNA: p<0.0001; TGF: n.s.)

	SM-Fig. 3E: Extracellular FN1 Relative Protein Leves (% of siNC-							
	Ctrl			TGF vs Ctrl	siAtg5/7			siRNA vs TGF
	Mean	*SD*	*n*	*pV (†)*	*Mean*	*SD*	*n*	*pV (‡)*
**Ctrl**	100.00	0.00	4		70.00	15.700	4	
**TGFβ1**	143.30	34.30	4	0.05	83.30	17.100	4	<0.01
**TGFβ2**	184.00	49.10	4	0.05	126.00	35.000	4	<0.05

(†) One-way ANOVA (p=0.02)
(‡) Two-Way Anova (siRNA: p=0.0008; TGF: p=0.0007)

	SM-Fig. 3F: Extracellular Col I Relative Protein Leves (% of siNC-							
	Ctrl			TGF vs Ctrl	siAtg5/7			siRNA vs TGF
	Mean	*SD*	*n*	*pV (†)*	*Mean*	*SD*	*n*	*pV (‡)*
**Ctrl**	100	0	4		80.3	4.7	4	
**TGFβ1**	143.66	25.1	4	0.05	110	11.9	4	n.s.
**TGFβ2**	153.3	48	4	n.s.	120.3	21.7	4	n.s.

(†) One-way ANOVA (p=n.s.)
(‡) Two-Way Anova (siRNA: p=0.01; TGF: p=0.003)

Figure 3-SM: Relative protein levels quantification of total LC3, Atg5, and Smad2/3 calculated from densitometric analysis of the bands (Figure 3A), expressed as percentage of $siNC_{Ctrl}$ (set to 100%). β-actin was used as loading control. two-way ANOVA with Bonferroni post-test. RPL: Relative protein levels.

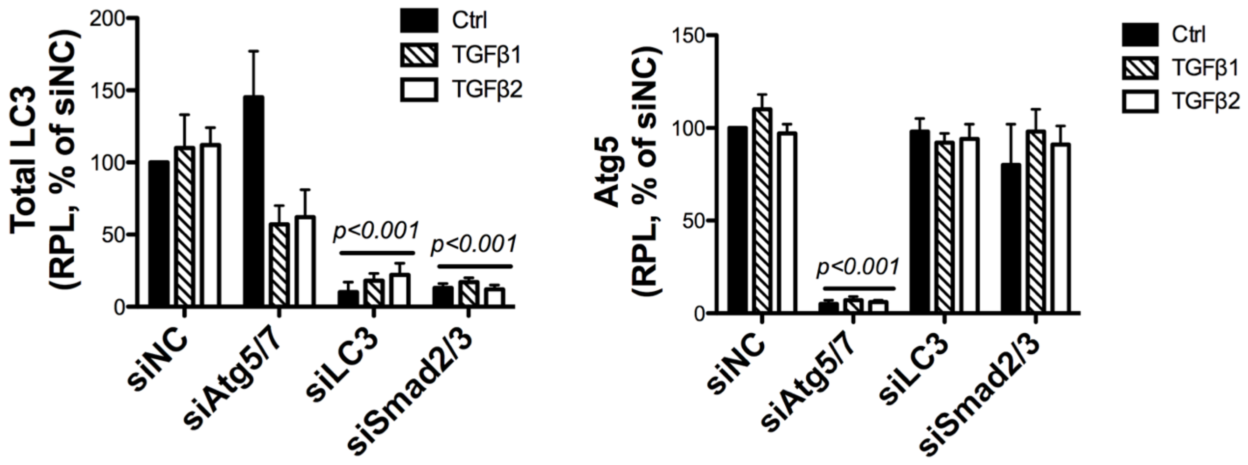

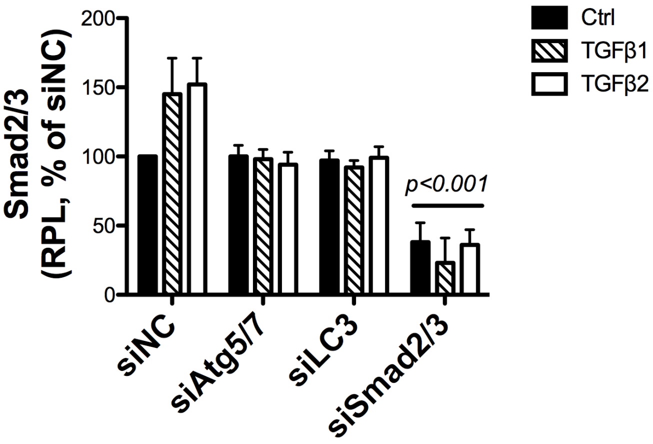
Smad2/3
(RPL, % of siNC)
200
150
100
50
0
p<0.001
siNC
siAtg5/7
siLC3
siSmad2/3
Ctrl
TGFβ1
TGFβ2

SUPLEMENTAL MATERIAL TO FIGURE 4

Exact mean values ±SD and statistical analysis

	SM-Fig. 4B: SMA Relative Protein Leves (% of siNC-Ctrl)											
	**Veh**			**TGF vs Veh**	**3-MA**			**Drug vs TGF**	**BafA1**			**Drug vs TGF**
	Mean	*SD*	*n*	*pV (†)*	*Mean*	*SD*	*n*	*pV (‡)*	*Mean*	*SD*	*n*	*pV (‡)*
**Ctrl**	100	0	3		47.6	11.3	3		28.66	12.42	3	
**TGFβ1**	199.7	65.7	3	0.05	82	20.6	3	<0.05	43.3	9.4	3	<0.01
**TGFβ2**	268	123	3	n.s.	108.3	34.7	3	<0.01	24.6	15.5	3	<0.0001

(†) *One-way ANOVA (p=n.s)*

(‡) *Two-Way Anova (Drug: p<0.0001; TGF: p=0.01)*

	SM-Fig. 4C: FN1 Relative Protein Leves (% of siNC-Ctrl)											
	**Veh**			**TGF vs Veh**	**3-MA**			**Drug vs TGF**	**BafA1**			**Drug vs TGF**
	Mean	*SD*	*n*	*pV (†)*	*Mean*	*SD*	*n*	*pV (‡)*	*Mean*	*SD*	*n*	*pV (‡)*
**Ctrl**	100	0	3		30	9.89	3		236	65.5	3	
**TGFβ1**	265	76.3	3	0.05	94	8.48	3	<0.05	568.5	115.2	3	<0.001
**TGFβ2**	212	106	3	n.s.	54.5	13.4	3	<0.05	362	53.7	3	n.s.

(†) *One-way ANOVA (p=0.05)*

(‡) *Two-Way Anova (Interaction: p=0.03; Drug: p<0.0001; TGF: p<0.0001)*

SUPLEMENTAL MATERIAL TO FIGURE 5

Exact mean values ±SD and statistical analysis

	SM-Fig. 5B: pSmad2/3 (% of siNC at t=0 post TGF treatment)							
	**Ctrl**			**TGF vs Ctrl**	**siAtg5/7**			**siRNA vs TGF**
	Mean	*SD*	*n*	*pV (†)*	*Mean*	*SD*	*n*	*pV (‡)*
**Ctrl**	100	0	3		95	7	3	
**15 min**	210	14.1	3	p<0.001	132.5	3.5	3	p<0.05
**30 min**	390	132	3	p<0.001	255	63.6	3	p<0.001

(†) *One-way ANOVA (p<0.0001)*

(‡) *Two-Way Anova (Interaction: p<0.005; siRNA: p=0.0001; TGF: p<0.0001)*

Figure 5-SM1 : Effectt of siBAMBI in Smad2/3 phosphorylation in control cells. Human TM primary cells were transfected with siNC, siBAMBI or siBAMBI and siATG5/7. At 2 d.p.t, cells were treated with TGFβ2 (10 ng/mL). Phosphorylated and total Smad2/3 protein levels were evaluated by western blot in whole cell lysates (5 μg). t-test, n=2.

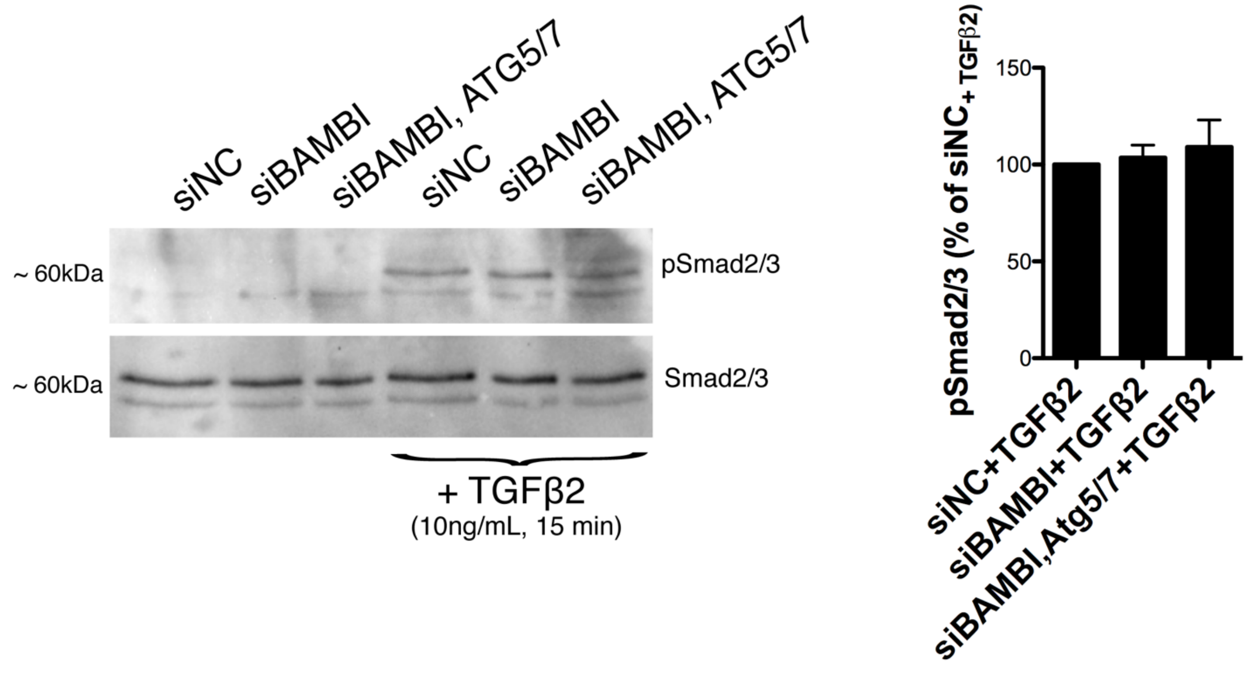

Figure 5-SM2 : Relative protein levels quantification of Atg5 and Smad2/3 calculated from densitometric analysis of the bands, expressed as percentage of $siNC_{t=0}$ or $siNC_{Ctrl}$. β-actin was used as loading control. two-way ANOVA with Bonferroni post-test. RPL: Relative protein levels.

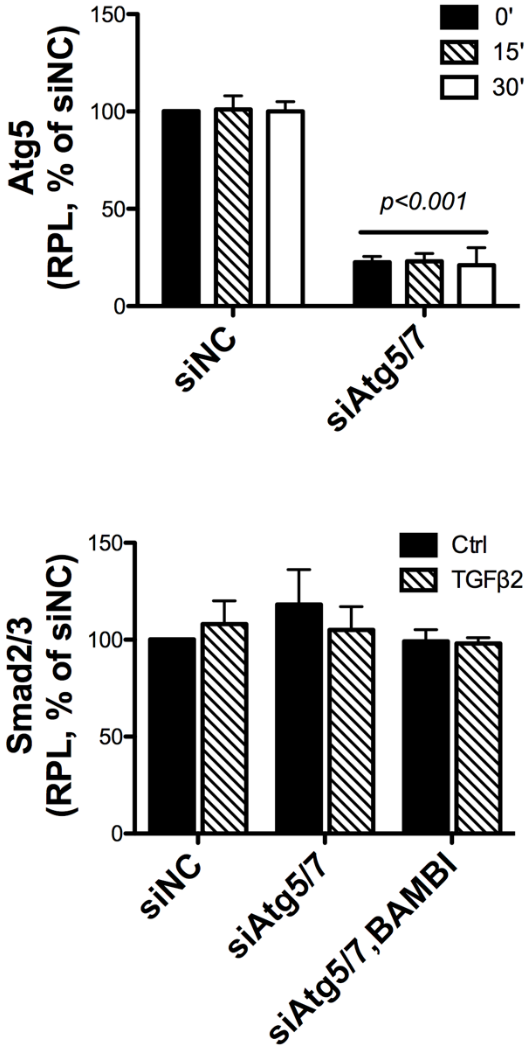

SUPLEMENTAL MATERIAL TO FIGURE 6

Exact mean values ±SD and statistical analysis

	SM-Fig.6C: LC3-II Relative Protein Leves (% of Veh-Ctrl)						
	Ctrl			TGF vs Ctrl	BafA1		
	Mean	*SD*	*n*	*pV (†)*	*Mean*	*SD*	*n*
**Ctrl**	100.00	0.00	3		247.00	35.00	3
**TGFβ1**	119.00	7.00	3	p<0.01	163.00	50.00	3
**TGFβ2**	124.00	6.00	3	p<0.01	123.00	22.00	3

(†) One-way ANOVA (p=0.0034)

	SM-Fig.6C: P62 Relative Protein Leves (% of Veh-Ctrl)						
	Ctrl			TGF vs Ctrl	BafA1		
	Mean	*SD*	*n*	*pV (†)*	*Mean*	*SD*	*n*
**Ctrl**	100.00	0.00	3		283	35	3
**TGFβ1**	48.20	15.00	3	p<0.01	233	12	3
**TGFβ2**	45.15	20.00	3	p<0.01	230	46	3

(†) One-way ANOVA (p=0.0057)

Figure 6-SM: Relative protein levels quantification of SMA calculated from densitometric analysis of the bands, expressed as percentage of Veh_{Ctrl}. β-actin was used as loading control. † denotes statistical significance when comparing TGFβ treatment versus control, using one-way ANOVA with multiple comparisons; ‡ denotes statistical significance when comparing BafA1-treated versus their respective control, using two-way ANOVA with Bonferroni post-test. RPL: Relative protein levels.

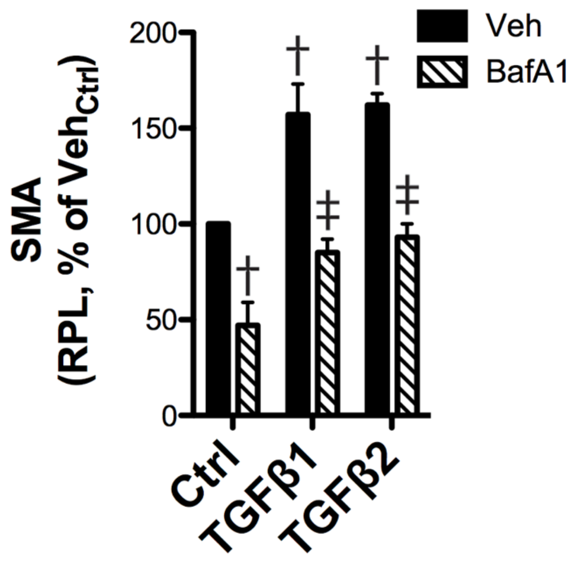

SUPLEMENTAL MATERIAL TO FIGURE 7

Exact mean values ±SD and statistical analysis

	SM-Fig.7B: LC3-II Relative Protein Leves (% of siNC-Ctrl)							
	siNC				siSmad2/3			
	Mean	*SD*	*n*	*pV (†)*	*Mean*	*SD*	*n*	*pV (‡)*
**Ctrl**	100	0	4		30	20	4	p<0.001
**TGFβ1**	139	19	4	0.05	13	13	4	p<0.0001
**TGFβ2**	158	32	4	0.01	32	16	4	p<0.0001

(†) *One-way ANOVA (p=0.011)*

(‡) *Two-Way Anova (Interaction: p=0.01; siRNA: p<0.0001; TGF: p=0.02)*

	SM-Fig.7C: LC3-I Relative Protein Leves (% of siNC-Ctrl)							
	siNC				siSmad2/3			
	Mean	*SD*	*n*	*pV (†)*	*Mean*	*SD*	*n*	*pV (‡)*
**Ctrl**	100	0	4		61	25	4	
**TGFβ1**	125	9	4	n.s	55	24	4	p<0.001
**TGFβ2**	143	23	4	0.010	37	30	4	p<0.0001

(†) *One-way ANOVA (p=0.006)*

(‡) *Two-Way Anova (Interaction: p=0.05; siRNA: p<0.0001; TGF: p=n.s.)*

Figure 7-SM: Relative protein levels quantification of Smad2/3 calculated from densitometric analysis of the bands, expressed as percentage of $siNC_{Ctrl}$. β-actin was used as loading control. Two-way ANOVA with Bonferroni post-test. RPL: Relative protein levels.

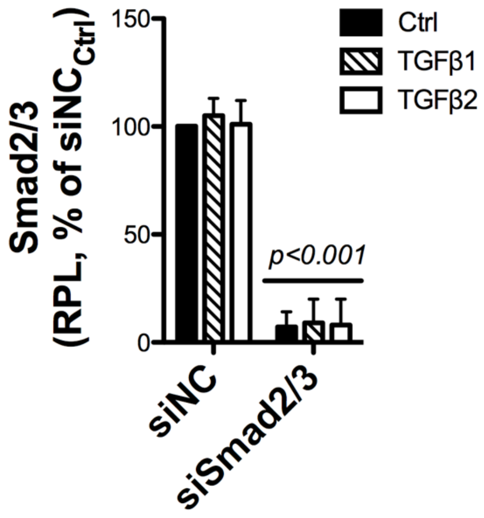

Smad2/3
(RPL, % of siNC$_{Ctrl}$)
150
100
50
0
Ctrl
TGFβ1
TGFβ2
p<0.001
siNC
siSmad2/3

Figure 3A- Full Length Western-blots

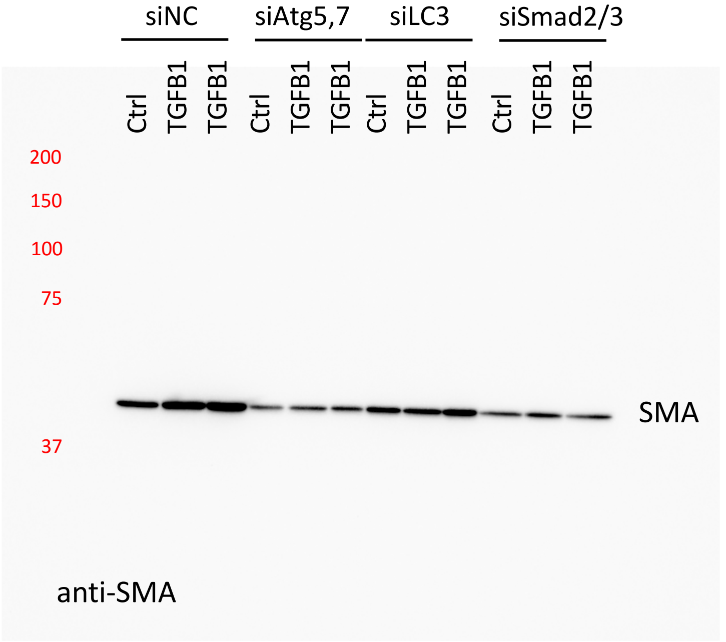

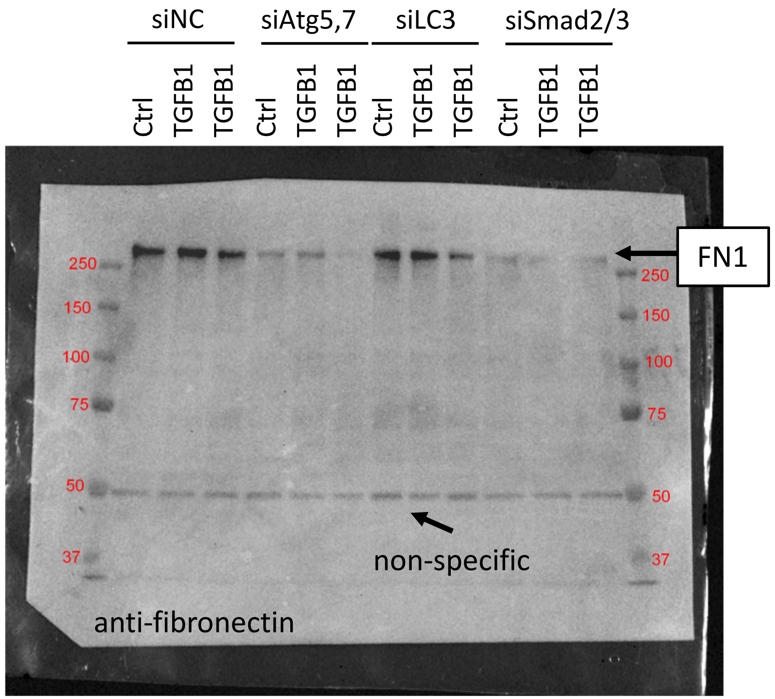

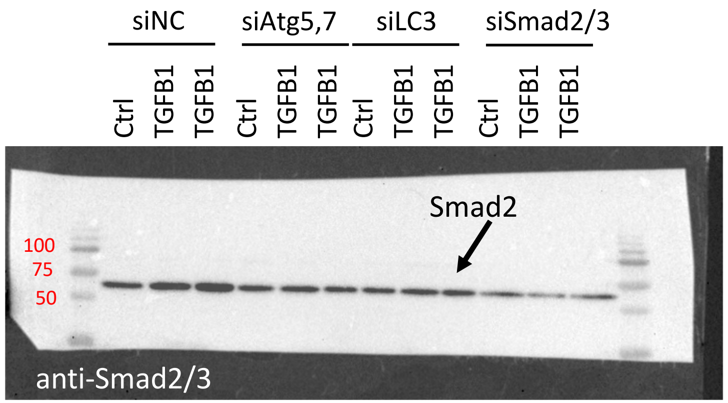

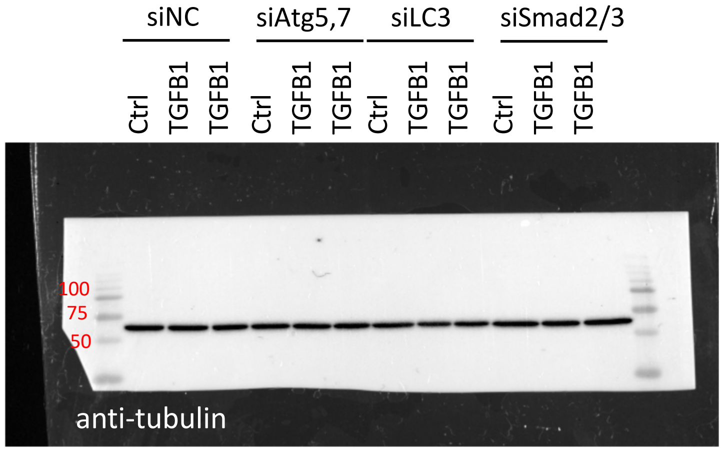

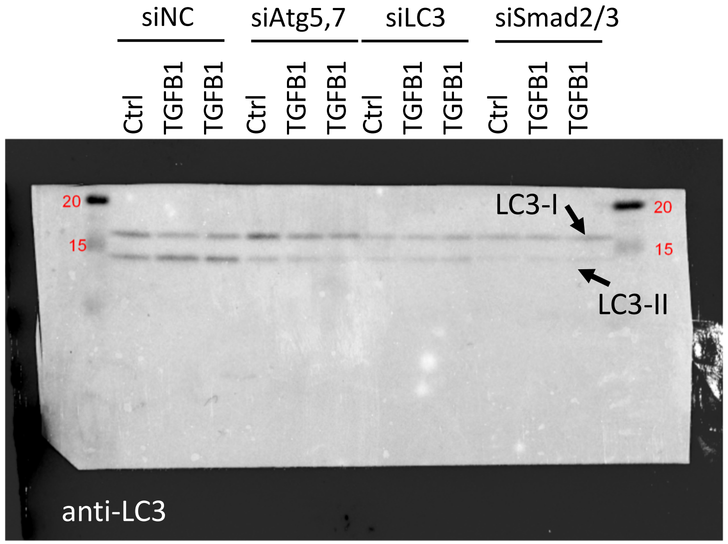

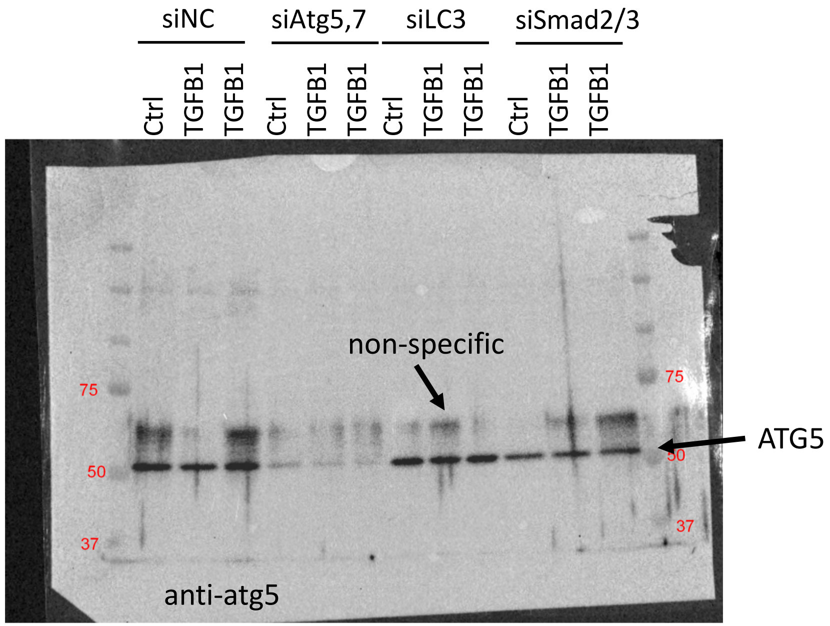

Figure 3D- Full Length Western-blots

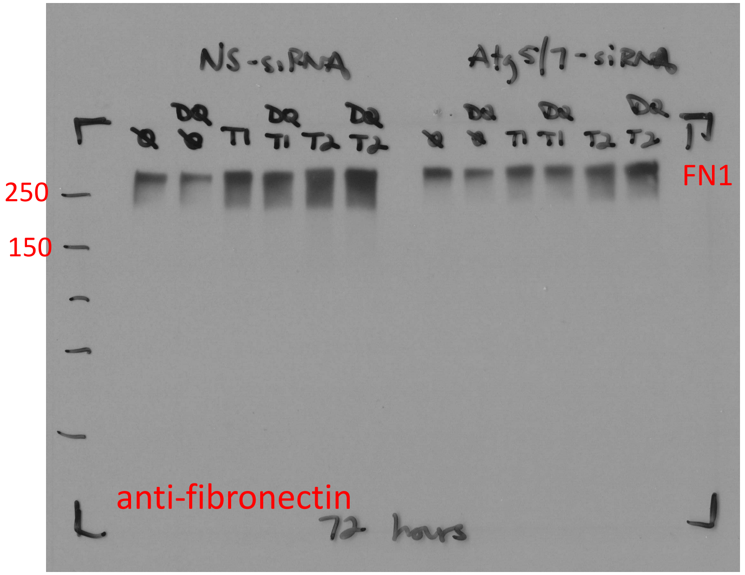

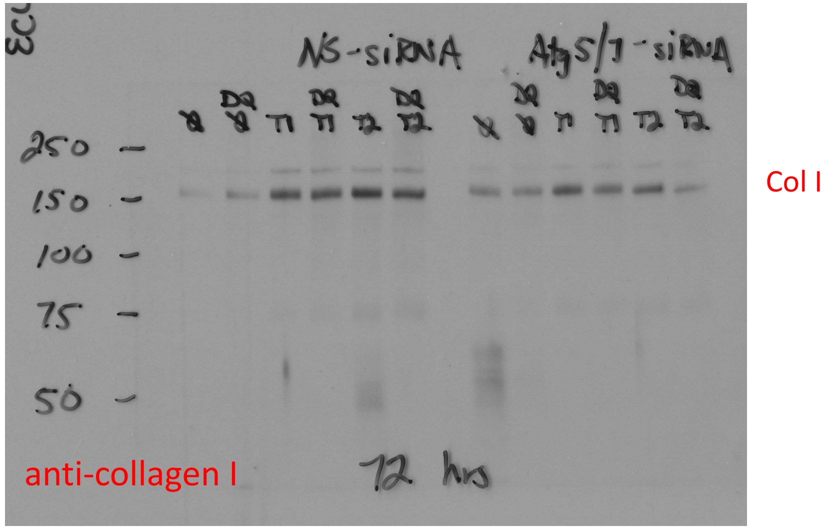

Figure 4- Full Length Western-blots

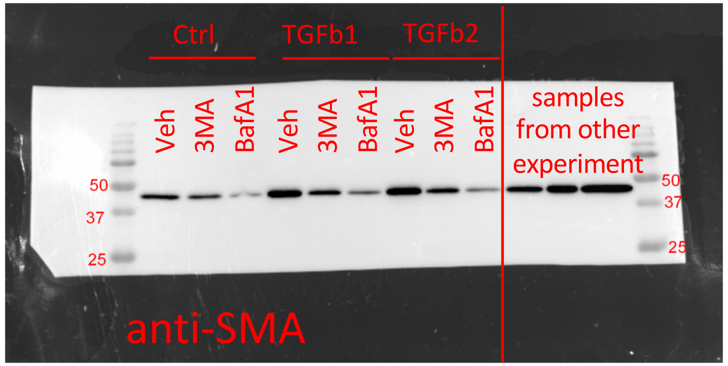

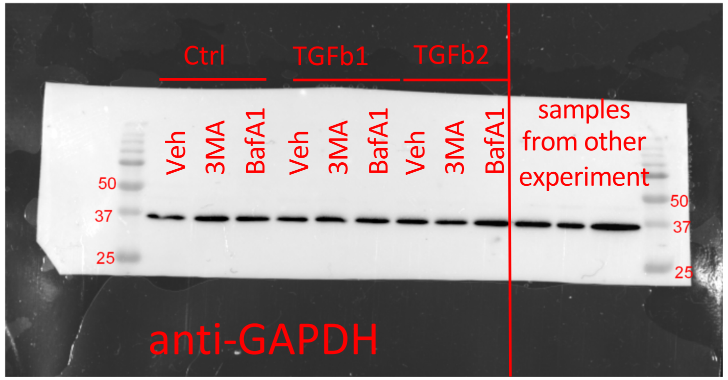

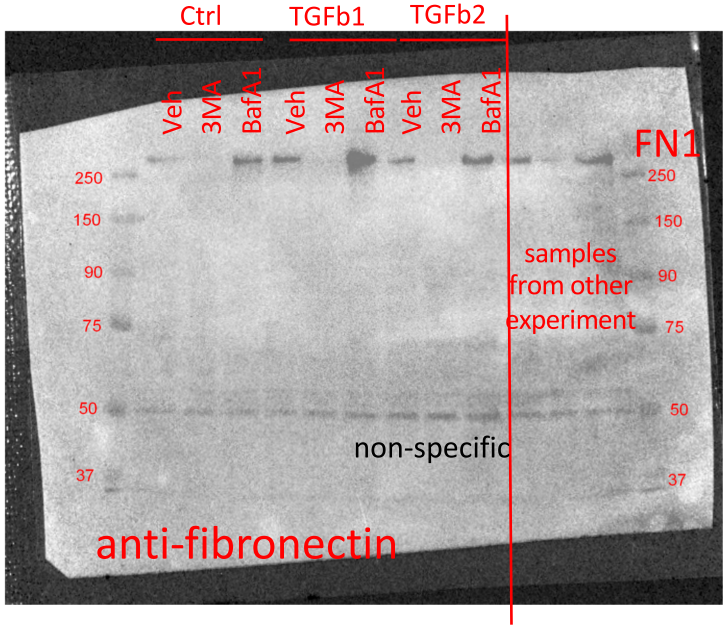

Figure 5- Full Length Western-blots

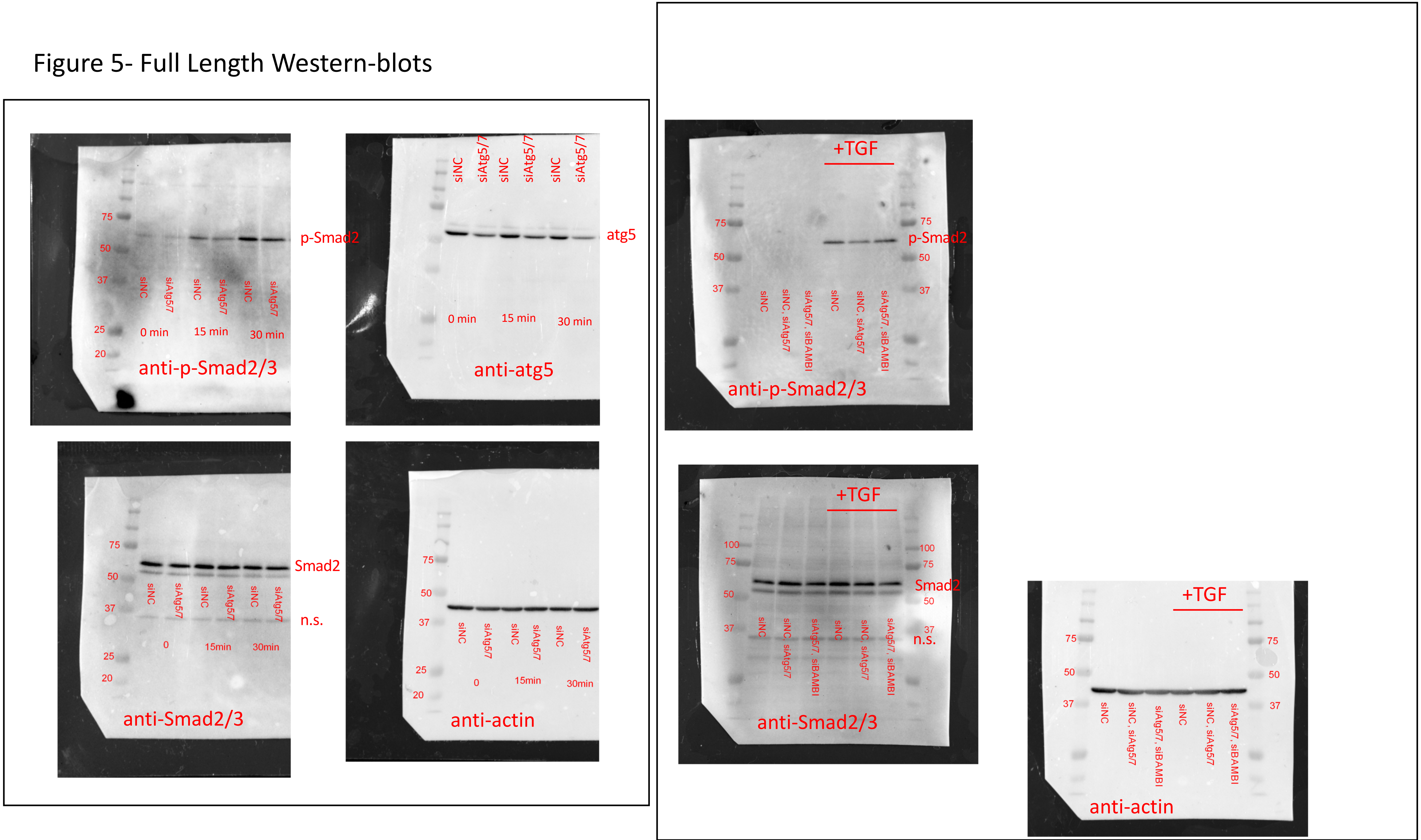

Figure 6- Full Length Western-blots

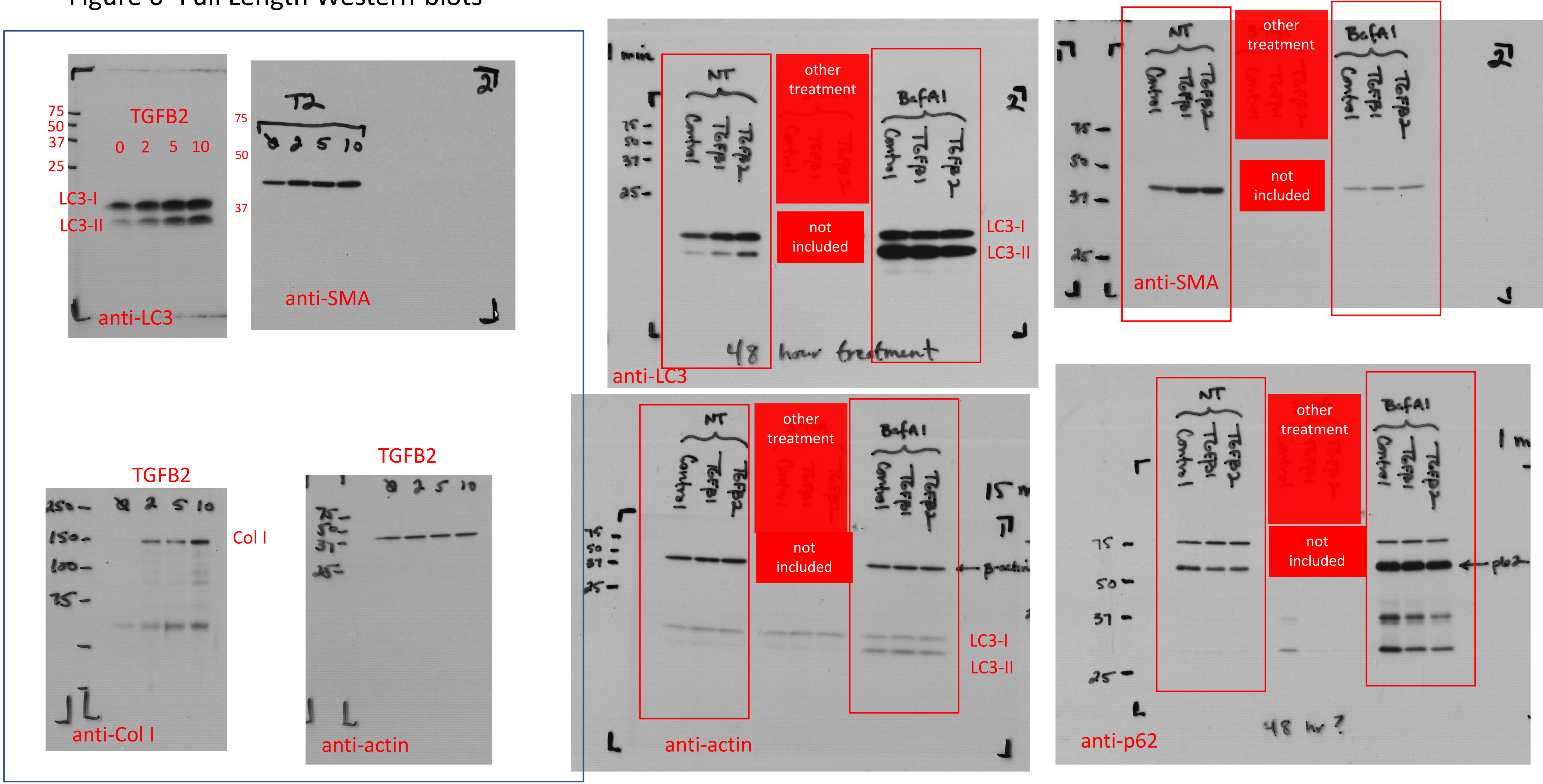

Figure 7- Full Length Western-blots

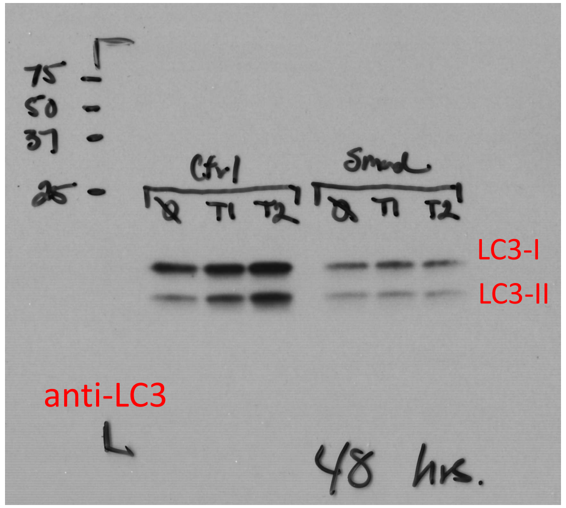

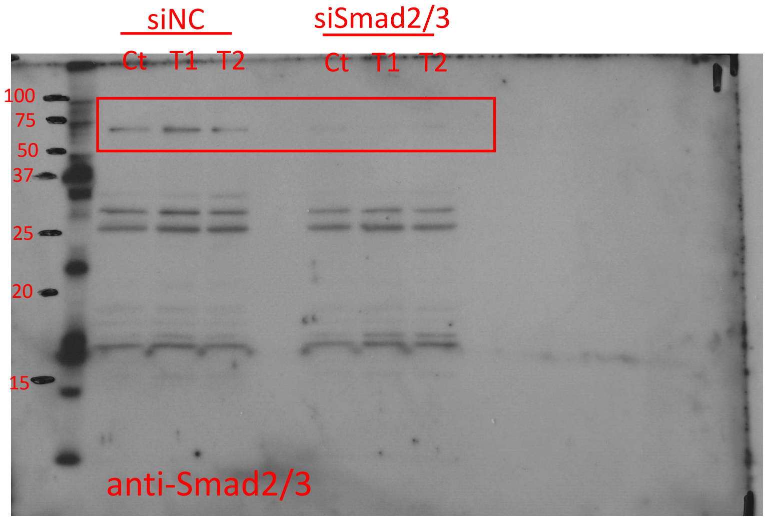

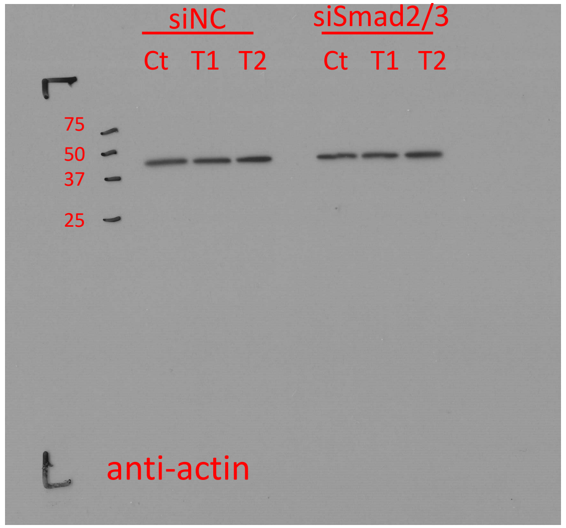

Supplemental Material for Figure 5

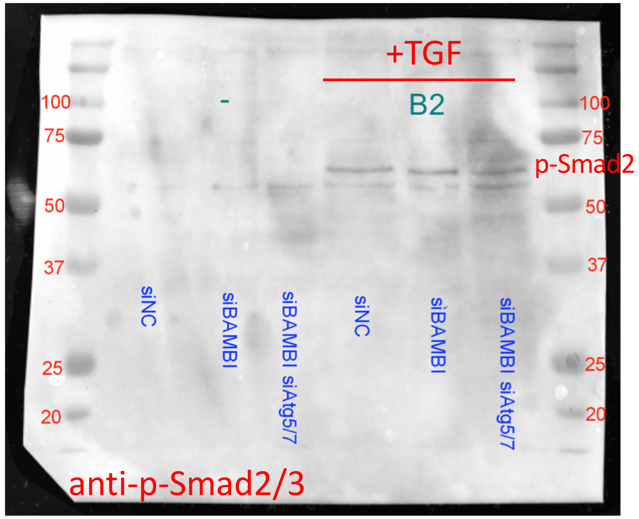

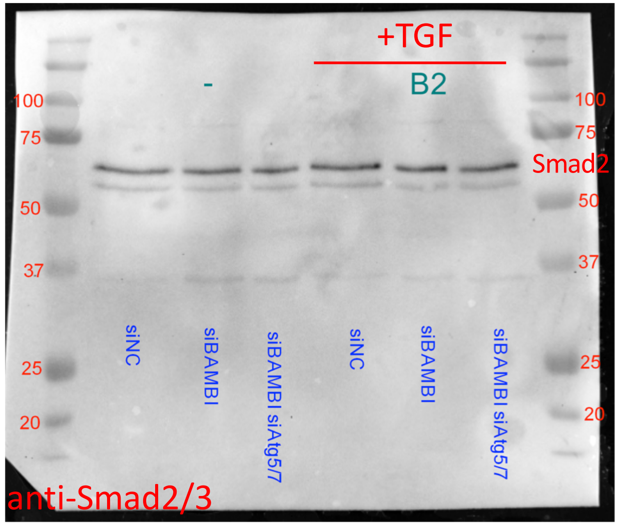

TEST ANTI-HUMAN BAMBI ANTIBODIES

BAMBI expected MW: 29 kDa

Bambi antibody
Abcam: ab200737, lot: GR3198849-1

Pic from Abcam

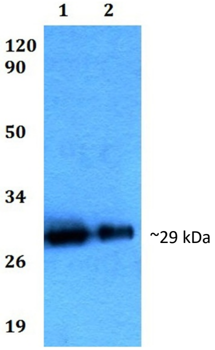

HTM

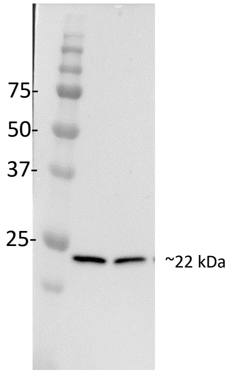

Protein previously labeled as BAMBI.

Only band detectable shows incorrect MW and it does not show lower levels with siBAMBI after 72h (confirmed by qPCR)

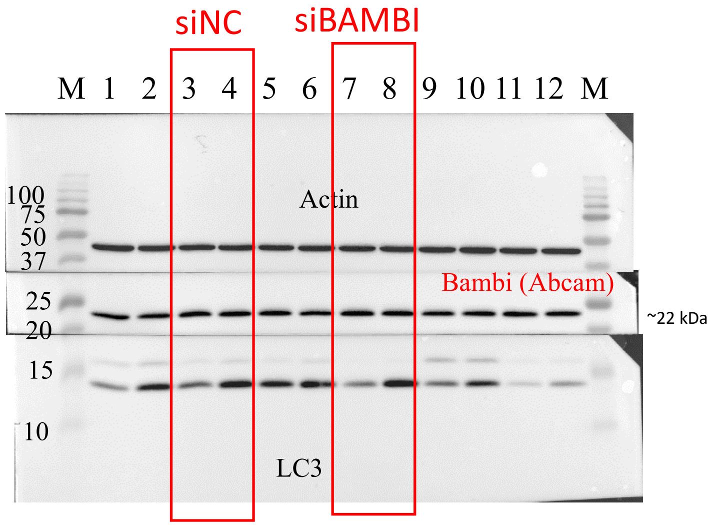

Bambi antibody
Novus (R&D system): AF921

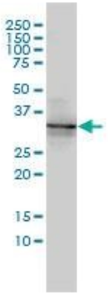

No clear band was detected at Bambi MW and no lower expression intensity was observed in any band in the siBAMBI cells. We tried also with 20 ug of protein lysate with no success.

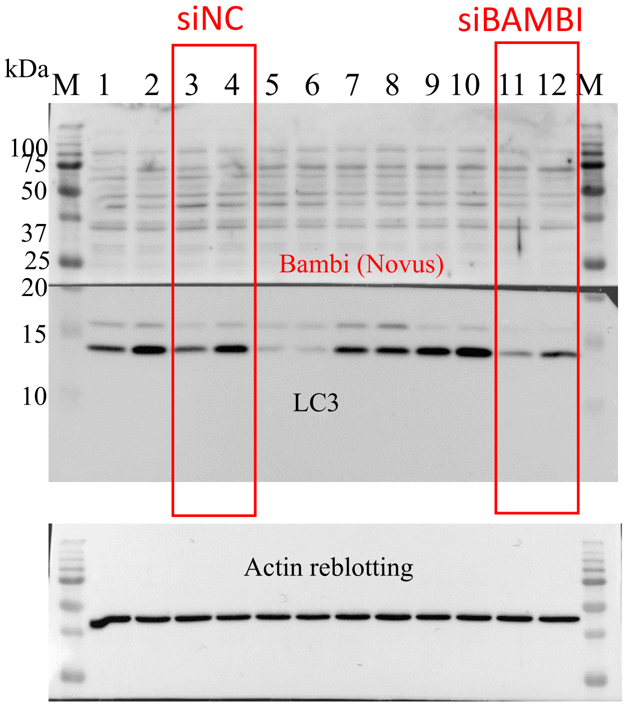

Bambi antibody
Santa Cruz: sc-100681

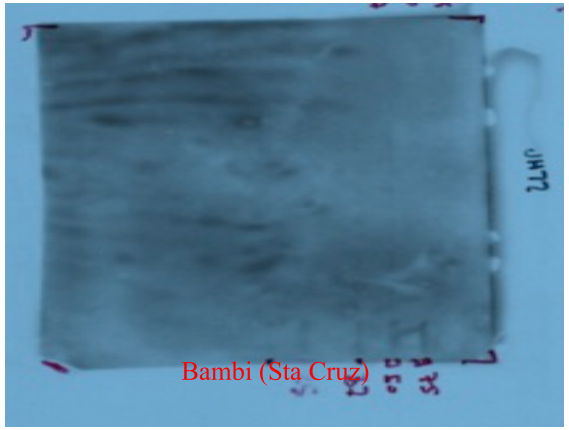

No band was detected after 30min exposure with ECL2. Notice appropriate protein content and transfer in actin blot.

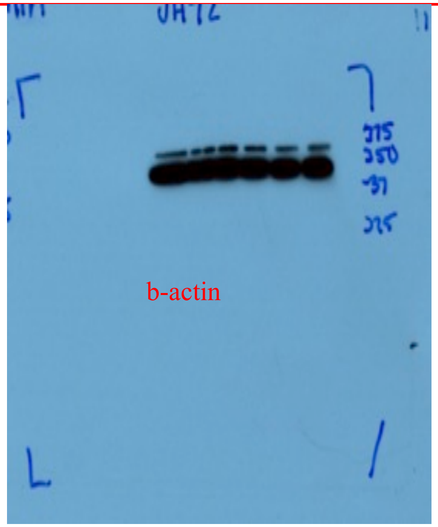

Supplement: Supplementary file 3 — Supplemental Material [file 41598_2019_52627_MOESM3_ESM.pdf]
